# GmNAC81 Inversely Modulates Leaf Senescence and Drought Tolerance

**DOI:** 10.3389/fgene.2020.601876

**Published:** 2020-11-24

**Authors:** Dalton O. Ferreira, Otto T. Fraga, Maiana R. Pimenta, Hanna D. N. Caetano, João Paulo B. Machado, Paola A. Carpinetti, Otávio J. B. Brustolini, Iana P. S. Quadros, Pedro A. B. Reis, Elizabeth P. B. Fontes

**Affiliations:** ^1^National Institute of Science and Technology in Plant-Pest Interactions, Bioagro, Universidade Federal de Viçosa, Viçosa, Brazil; ^2^Department of Biochemistry and Molecular Biology/BIOAGRO, Universidade Federal de Viçosa, Viçosa, Brazil; ^3^Núcleo de Graduação de Agronomia, Universidade Federal de Sergipe, Nossa Senhora da Glória, Brazil; ^4^Agronomy Institute, Universidade Federal de Viçosa, Florestal, Brazil; ^5^National Laboratory for Scientific Computing (LNCC), Petrópolis, Brazil

**Keywords:** GmNAC81, *Glycine max*, leaf senescence, drought tolerance, NAC transcription factors

## Abstract

*Glycine max* NAC81 (GmNAC81) is a downstream effector of the DCD/NRP-mediated cell death signaling, which interacts with GmNAC30 to fully induce the caspase 1-like vacuolar processing enzyme (VPE) expression, the executioner of the cell death program. GmNAC81 has been previously shown to positively modulate leaf senescence via the NRP/GmNAC81/VPE signaling module. Here, we examined the transcriptome induced by *GmNAC81* overexpression and leaf senescence and showed that GmNAC81 further modulates leaf senescence by regulating an extensive repertoire of functionally characterized senescence-associated genes (SAGs). Because the NRP/GmNAC81/VPE signaling circuit also relays stress-induced cell death signals, we examined the effect of *GmNAC81* overexpression in drought responses. Enhanced *GmNAC81* expression in the transgenic lines increased sensitivity to water deprivation. Under progressive drought, the *GmNAC81*-overexpressing lines displayed severe leaf wilting, a larger and faster decline in leaf Ψw, relative water content (RWC), photosynthesis rate, stomatal conductance, and transpiration rate, in addition to higher Ci/Ca and lower Fm/Fv ratios compared to the BR16 control line. Collectively, these results indicate that the photosynthetic activity and apparatus were more affected by drought in the transgenic lines. Consistent with hypersensitivity to drought, chlorophyll loss, and lipid peroxidation were higher in the *GmNAC81*-overexpressing lines than in BR16 under dehydration. In addition to inducing VPE expression, *GmNAC81* overexpression uncovered the regulation of typical drought-responsive genes. In particular, key regulators and effectors of ABA signaling were suppressed by *GmNAC81* overexpression. These results suggest that GmNAC81 may negatively control drought tolerance not only via VPE activation but also via suppression of ABA signaling.

## Introduction

The NAC (an acronym for NAM, ATAF, and CUC) superfamily of plant-specific transcriptional factors (TF) is largely distributed in the plant kingdom and constitutes one of the largest families of plant TFs (Shao et al., 2015). The primary structural organization of NAC TFs includes a 150-aminoacid conserved N-terminal DNA binding domain, designated the NAC domain, and a more variable C-terminal domain involved in transcriptional regulation (Nakashima et al., 2011). The NAC domain is divided into five subdomains (A-E). The C and D subdomains are highly conserved and are responsible for binding DNA. In contrast, the most divergent B and E subdomains may be responsible for the functional diversity of NAC genes, whereas the A subdomain may be involved in the formation of functional homo- or heterodimers or oligomers (Puranik et al., 2012). The D subdomain of some NACs may harbor a highly hydrophobic, negative regulatory domain (NRD), which inhibits NAC and TF partners (Puranik et al., 2012).

NAC proteins are mediators of responses to a range of developmental and environmental signals. Functionally characterized NACs display essential roles in flower development (Sablowski and Meyerowitz, 1998), apical meristem formation (Souer et al., 1996; Aida et al., 1997; Hibara et al., 2003), hormone signaling (Xie et al., 2000; Fujita et al., 2004), regulation of secondary cell wall biosynthesis (Mitsuda and Ohme-Takagi, 2008; Negi et al., 2017), regulation of leaf senescence (Kim et al., 2016), cell cycle control (Kim et al., 2006), lateral root development (Hao et al., 2011), pathogen infection responses (Collinge and Boller, 2001; Selth et al., 2005) and abiotic stress responses (Tran et al., 2004, 2010; Fang et al., 2008; Tweneboah and Oh, 2017; Chung et al., 2018). Therefore, the plant NAC superfamily relevance is incontestable, but functional studies of NAC genes in crop species are still limited.

In soybean, the superfamily encompasses 182 genes distributed evenly in the soybean chromosomes (Melo et al., 2018). Some NAC proteins are involved in both development and stress responses. Examples are GmNAC81 and GmNAC30, which form heterodimers to activate a cell death program via the upregulation of the caspase1-like vacuolar processing enzyme (VPE) expression (Hara-Nishimura et al., 2011; Mendes et al., 2013). Both GmNAC81 and GmNAC30 are coordinately regulated in response to several different stresses, cell death inducers, hormone signals, and developmentally programmed leaf senescence (Carvalho et al., 2014b; Pimenta et al., 2016).

GmNAC81 was first identified as a downstream component of the developmental cell death (DCD) domain-containing Asparagine (N)-rich protein, DCD/NRP-mediated cell death response (Faria et al., 2011; Reis et al., 2011; Reis and Fontes, 2012). This pathway has been described as a convergent response of osmotic and endoplasmic reticulum stress (Irsigler et al., 2007; Costa et al., 2008). The combination of both treatments promotes a synergistic effect in the *DCD/NRPs* expression mediated by the stress-induced transcriptional factor GmERD15 (Irsigler et al., 2007; Alves et al., 2011). The accumulation of DCD/NRP activates a signaling cascade leading to the upregulation of *GmNAC81* and *GmNAC30* (Mendes et al., 2013). In the nucleus, these TFs form a heterodimer that binds to a specific cis-regulatory element (TGTG[T/C/G]) on the VPE promoter to activate *VPE* expression, the executioner of a plant-specific programmed cell death (PCD) mediated by the collapse of the vacuole (Hara-Nishimura et al., 2011). In addition to VPE, the repertoire of GmNAC81 and GmNAC30 upregulated genes includes predominantly hydrolytic enzymes (carboxypeptidase, phosphatase, and glucanase) that may also be involved in plant PCD (Mendes et al., 2013). GmNAC81 has also been implicated as a positive modulator or developmentally programmed leaf senescence (Pimenta et al., 2016). Therefore, the NRP/GmNAC81:GmNAC30/VPE signaling module may function as a regulatory circuit that integrates developmentally programmed leaf senescence with stress-induced cell death. To gain insights into these linked responses’ intricacy, we ectopically expressed *GmNAC81* in soybean and analyzed the overlapping and specific features of these responses. We show here that in addition to promoting stress-induced cell death, GmNAC81 directly regulates drought-responsive genes and increases drought sensitivity.

## Methods

A detailed description of methods is provided in Supplementary Methods.

Briefly, GmNAC81–1 and GmNAC81–3 lines have been previously described (Pimenta et al., 2016). RNA sequencing and differential gene expression analyses were performed according to Melo et al. (2018). RNA-sequencing data were deposited in the Gene Expression Omnibus under accession number GSE159910^1^. Physiological parameter measurements were carried out as described (Pimenta et al., 2016), and water stress induction and monitoring were conducted according to Carvalho et al. (2014a).

## Results

### *GmNAC81* Ectopic and Endogenous Expression Equalizes During the Onset of Leaf Senescence

**FIGURE 1:**
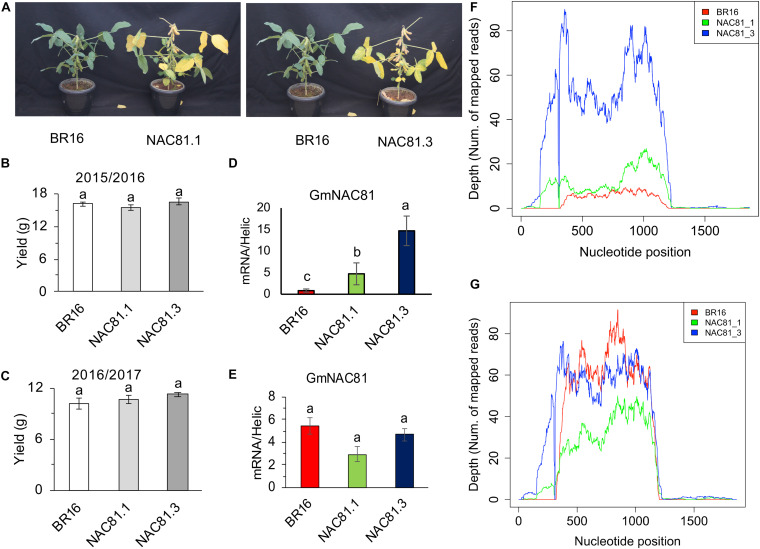
*GmNAC81* overexpression accelerates leaf senescence. **(A)** Phenotype of *GmNAC81*-overexpressing lines and the cultivar BR16 at 90 days after germination (DAG). **(B)** Mass of grains of BR16 and GmNAC81 lines in the first growing season (2015/2016) (mean ± standard error). Means followed by different letters are significantly different by *t*-test (*p* < 0.05). **(C)** Mass of grains of BR16 and GmNAC81 lines in the second growing season (2016/2017) (mean ± standard error). Means followed by different letters are significantly different by *t*-test (*p* < 0.05). **(D)** Normalized expression of *GmNAC81* at 20 DAG. The accumulation of transcripts of *GmNAC81* was determined by 2^–ΔΔCt^ (mean ± standard error). Means followed by different letters are significantly different by *t*-test (*p* < 0.05). **(E)** Normalized expression of *GmNAC81* at 80 DAG. The accumulation of the GmNAC81 transcripts was determined by 2^–ΔΔCt^ (mean ± standard error). Means followed by different letters are significantly different by *t*-test (*p* < 0.05). **(F)** High throughput sequencing reads mapped to the GmNAC81 locus (Glyma.12G022700, Phytozome 12) at 20 DAG. **(G)** High throughput sequencing reads mapped to the GmNAC81 locus at 80 DAG.

The transgenic lines GmNAC81–1 and GmNAC81–3 have been fully described previously (Pimenta et al., 2016). Here, we further complemented these previous studies by demonstrating first that despite accelerating leaf senescence (Figure 1A; Pimenta et al., 2016), *GmNAC81* overexpression does not cause yield penalty.

The *GmNAC81*-overexpressing lines displayed accelerated leaf senescence (Figure 1A). At 90 days after germination (DAG), developmental stage R7, leaf yellowing was more intense in transgenic lines than in wild type (Figure 1A), which was associated with the higher accumulation of hydrogen peroxide in overexpressing lines, as judged by the intensity of DAB staining in senescent leaves (Supplementary Figure S1A). The precocious leaf senescence phenotype of the transgenic lines (Figure 1A) confirmed that the GmNAC81 transgene remained functional in the R5 generations of GmNAC81–1 and GmNAC81–3 lines, which were used for RNA-sequencing. Very importantly, the seed yield of *GmNAC81*-overexpressing lines was similar to that of the wild type (Figures 1B,C). The lower and greater pod numbers displayed by GmNAC81–1 and GmNAC81–3 lines, respectively, were compensated by differences in the weight of 1,000 seeds among genotypes in the growing seasons 2015/2016 and 2016/2017 (Supplementary Figures S1B,C). Therefore, seed weight/plant was similar in transgenic lines and wild type, indicating that *GmNAC81* overexpression did not compromise yield (Figures 1B,C).

*GmNAC81* is positively regulated during leaf senescence (Carvalho et al., 2014b; Pimenta et al., 2016). The RNA-seq data confirmed a strong upregulation of *GmNAC81* during leaf senescence (Figures 1F,G). At 20 DAG (V3), *GmNAC81* expression was significantly higher in GmNAC81–3 and slightly higher in GmNAC81–1 lines compared to BR16 (Figure 1D and Supplementary Figure S1F). In contrast, at 80 DAG (R6), endogenous *GmNAC81* in the wild type was strongly induced, reaching a similar plateau as in the transgenic lines GmNAC81–3 and GmNAC81–1 (Figure 1G). These results were further confirmed by RT-qPCR, indicating that during the onset of leaf senescence, *GmNAC81* ectopic expression in transgenic lines was similar to the endogenous expression in the wild-type, BR16 line (80 DAG; Figure 1E). Therefore, to delimitate the GmNAC81-induced changes, we performed RNA-seq of GmNAC81–3 and BR16 leaves and compared the transcriptome changes in V3 leaves of GmNAC81_20DAG(V3) vs. BR16_20DAG(V3). Leaf senescence-induced changes were derived from both contrasts [BR16_80DAG(R6) vs. BR16_20DAG(V3)] and [GmNAC81_80DAG(R6) vs. GmNAC81_20DAG(V3)]. In the first contrast, we examined the effects of leaf senescence and GmNAC81 enhanced expression, and in the latter contrast, we analyzed leaf senescence-induced changes in GmNAC81 lines.

A global cluster analysis of the expressed sequences among BR16_20DAG leaves, BR16_80DAG leaves, GmNAC81_20DAG leaves, and GmNAC81_80DAG leaves revealed that the senescence-induced transcriptome of BR16 and GmNAC81 leaves at 80 DAG were most closely related; these samples clustered together with a high bootstrap probability and a high approximately unbiased *P*-value (Supplementary Figure S2A). These results confirmed that the effect of *GmNAC81* overexpression is compensated by the strong induction of the endogenous gene during the onset of natural senescence (BR16 at 80 DAG), leading to similar responses during senescence. However, at 20 DAG, the *GmNAC81*-induced transcriptome was similar to the transcriptomes represented by the senescence-induced transcriptome cluster and differed considerably from the BR16_20DAG transcriptome. These results indicated that the *GmNAC81*-mediated response and senescence-induced response shared similar mechanisms.

### *GmNAC81* Overexpression Accelerates Leaf Senescence via the Induction of VPE and Senescence-Associated Genes

GmNAC81 has been characterized as a downstream component of the DCD/NRP-mediated cell death response, induced by leaf senescence (Carvalho et al., 2014b; Pimenta et al., 2016). By using a corrected *p* < 0.05 and log2-fold-change > 1 or log2-fold-change < 1 criteria for differentially regulated genes, our RNA-seq data confirmed that all previously described components of DCD/NRP-mediated cell death signaling (*GmERD15*, *NRPs*, *GmNAC81*, *GmNAC30*, and γ*VPE*) were induced by leaf senescence (Supplementary Figure S3A). These results served as a validation criterion of our RNA-seq data. In the *GmNAC81*-overexpressing V3 leaves, the upstream components of GmNAC81 were not differentially expressed, except for *ERD15*, which was slightly downregulated [GmNAC81_20DAG(V3) vs. BR16_20DAG(V3)] (Supplementary Figure S3C). In contrast, two copies of *VPE*, which is a direct target of GmNAC81, were strongly induced, which may explain at least in part the accelerated senescence phenotype of the transgenic lines (Mendes et al., 2013; Carvalho et al., 2014b; Pimenta et al., 2016).

In the SNAC-A subfamily, GmNAC30 is most related to the Arabidopsis ATAF2 paralog and forms a cluster with GmNAC18 and GmNAC39 genes, which were induced by leaf senescence (Supplementary Figure S4). In contrast, the other four SNAC-A genes were repressed by leaf senescence (Supplementary Figure S3A); they are more divergent from GmNAC30 and clustered together with ATAF1 in a phylogenetic analysis (Supplementary Figure S4). GmNAC30 has been previously shown to interact with GmNAC81 to fully induce γVPE Glyma.14G092800 (Mendes et al., 2013). RNA-seq also demonstrated that the paralog γVPE Glyma.17G230700 might be a direct target of GmNAC81 and GmNAC30. Accordingly, at the V3 stage (20 DAG), *GmNAC81* overexpression in the transgenic leaves also induced both Glyma.14G092800 and Glyma.17G230700 VPEs, although to a lesser extent compared to their expression at 80 DAG (Supplementary Figures S3A–C). The γVPE paralog Glyma.04G049900 was also induced by leaf senescence (80 DAG) but not by overexpressing *GmNAC81* in V3 leaves. These results are consistent with previous data showing that GmNAC81-regulated promoters also require GmNAC30 for full activation. In V3 leaves of *GmNAC81*-overexpressing lines, *GmNAC30* was not induced in parallel to provide a stoichiometric balance to the transcriptional activator complex.

To further validate the RNA-seq data, we examined the differential expression of other previously described GmNAC81 target genes (Mendes et al., 2013) in the contrast GmNAC81_20DAG(V3) vs. BR16_20DAG(V3). Four upregulated and two downregulated genes by the complex GmNAC81:GmNAC30 were correctly induced and repressed by *GmNAC81* overexpression in V3 leaves (Supplementary Figure S3D). These results further confirmed that the 35S:GmNAC81 transgene was functional.

**FIGURE 2:**
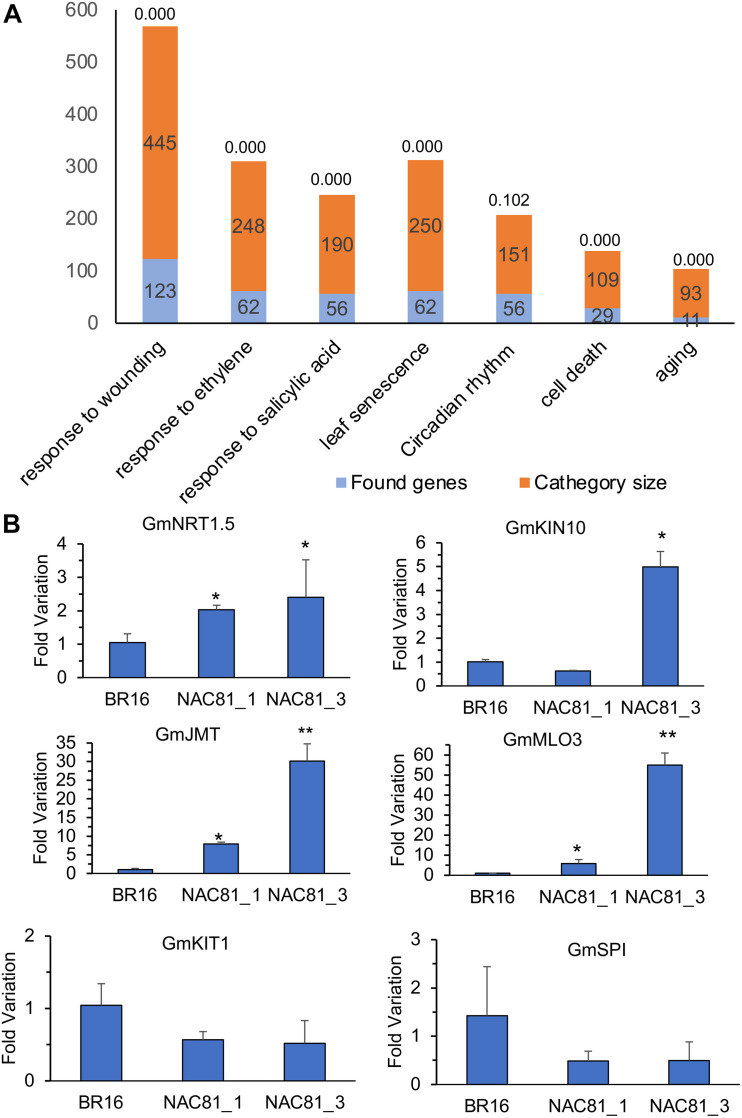
**(A)** Enrichment analysis of differentially expressed genes by *GmNAC81* overexpression [GmNAC81 (20DAG)–BR16 (20 DAG)] in their respective category of senescence-related GO terms. **(B)** Expression of candidate genes in soybean plants at 20 DAG. The accumulation of transcripts was determined by RT-qPCR (mean ± standard error). The Unknown 2 gene was used as an endogenous control to determine the Fold variation (2^–ΔΔ*Ct*^) of the respective genes *GmJMT*, *GmMLO3*, *GmKIN10*, *GmNRT1.5*, *GmSPI*, and *GmKIT1*. Significant results are indicated by *P < 0.05, **P < 0.01 compared to control (BR16).

To examine the transcriptional landscape of GmNAC81 during senescence, we compared the *GmNAC81*-induced transcriptome [GmNAC81_20DAG vs. BR16_20DAG] with the transcriptome induced by natural leaf senescence in BR16 [BR16_80DAG vs. BR16_20DAG] (Supplementary Figures S2B,C). Among the differentially expressed genes, 25.3% of upregulated genes and 42.7% of downregulated genes by *GmNAC81* overexpression at 20 DAG were also differentially regulated during the onset of natural senescence at 80 DAG. To gain further insights into the cellular processes affected by GmNAC81, we performed functional enrichment analyses of the differentially expressed genes identified in the contrast [GmNAC81_20DAG vs. BR16_20DAG]. Significantly enriched GO terms (with *p* < 0.05) were identified in all three categories, Biological Process, Molecular Function, or Cellular Component ontology. Consistent with the function of GmNAC81 in senescence, a subset of DEGs was significantly over-represented under senescence-related GO terms (Figure 2A and Supplementary Tables S1, S2).

Under the leaf senescence-enriched class, we selected a subset of induced and repressed genes for confirmatory analysis of gene expression by RT-qPCR (Supplementary Table S3). The upregulated senescence-associated genes included *NTR1.5* (Nitrate Transporter 1.5), *KIN10*, a catalytic subunit of SnRK1 (sucrose non-fermenting 1-related kinase 1 homolog 10) (Kim et al., 2016), *MLO3* (seven-transmembrane domain mildew resistance locus O3 protein (Piffanelli et al., 2002), and the wounding-induced *JMT* (Jasmonate O-methyltransferase) (Turner et al., 2002). NTR1.5 is a transmembrane protein responsible for nitrate transport, strongly induced by leaf senescence (Diaz et al., 2005, 2008; Fan et al., 2009). The selected KIN10 interacts directly and phosphorylates ETHYLENE INSENSITIVE 3, destabilizing an essential TF involved in ethylene signaling and senescence (Kim et al., 2016). Therefore, KIN10 negatively regulates ethylene signaling and hence may explain at least in part the massive downregulation of ethylene-responsive genes as a result of *GmNAC81* overexpression (Supplementary Table S2). In particular, *MLO3* was strongly induced by *GmNAC81* overexpression at 20 DAG and natural leaf senescence. MLO3 belongs to the plant-specific Mlo family of proteins with seven transmembrane segments and modulates defense- and environmentally induced cell death (Devoto et al., 1999; Piffanelli et al., 2002). The wounding-induced JMT promotes JA methylation to the activated form MeJA (Turner et al., 2002) and accelerates leaf senescence (Chena et al., 2017).

Among the downregulated genes, we selected *KIT1* (Kunitz trypsin inhibitor 1), induced by wounding and involved in programmed cell death (PCD) (Li et al., 2008, 2012) and *SPI* (serine protease inhibitor), a regulator of PCD (Clemente et al., 2019). The results of RT-qPCR confirmed the RNA-seq data. At 20 DAG, *NRT1.5*, *MLO3*, *JMT*, and *KIN10* genes were induced by enhanced expression of *GmNAC81*, whereas the *KIT1* and *SPI* genes were repressed (Figure 2B). The level of induction and repression of these representative genes correlated with transgene expression levels in the independently transformed lines GmNAC81–1 and GmNAC81–3 (Figure 1D). Furthermore, the promoters of the selected genes harbor variable numbers of GmNAC81 *cis*-regulatory elements [TGTG(TGC)]. The positions of GmNAC81 binding sites on the respective promoter and probability against random genomic sequences are shown in Supplementary Table S4. There are five repetitions of the GmNAC81 DNA binding site on the 5′ flanking sequences of NTR1.5 with a probability of 0.004 against random genomic sequence, indicating that *NTR1.5* is a potential target of GmNAC81. KIN10 promoter harbors three repetitions, MLO3 and KIT1 promoters, two repetitions. MLO3 and JMT promoters contain one single GmNAC81 binding site. Collectively, these data suggest that the selected senescence-related genes are potential targets of GmNAC81.

### *GmNAC81* Overexpression Increases Sensitivity to Drought Tolerance

**FIGURE 3:**
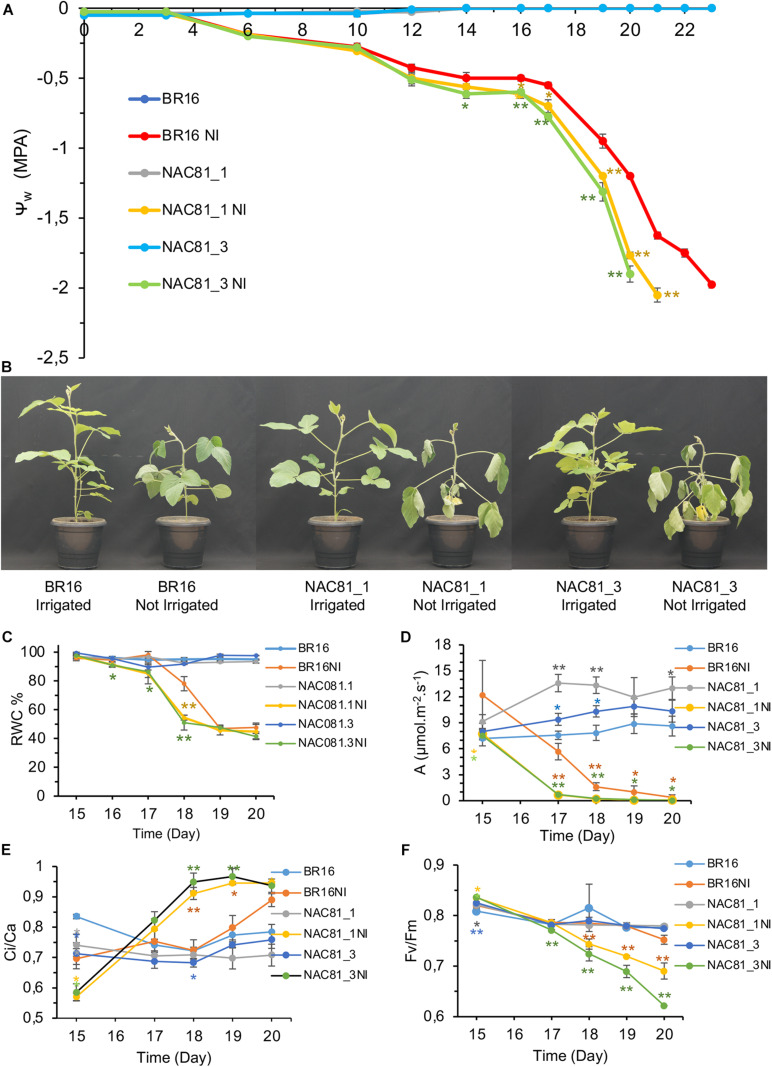
Soybeans plants in V3 stage submitted to a progressive water deficit regime. Drought stress was induced by a gradual reduction of the daily normal water supply. **(A)** Leaf water potential was measured with a Scholander pump over time. Numbers are days after the exposition to the water deficit regime. **(B)** Phenotype of soybean plants when the water potential reached −1.0 MPa in unirrigated BR16. **(C)** Relative water content of time. **(D)** Physiological parameters of gas exchange along the gradual drought stress Photosynthesis **(D,E)** Ci/Ca ratio. **(F)** Fluorescence physiological parameter, Fv/Fm ratio, throughout the gradual drought stress. The bars represent the standard error (*n* = 4). Significant results are indicated by *P < 0.05, **P < 0.01, compared to BR16 control lines. NI indicated not irrigated.

The negative modulation of the DCD/NRP-mediated cell death signaling enhances tolerance to drought (Carvalho et al., 2014a; Reis et al., 2016). Ectopic expression of the molecular chaperone binding protein (BiP) inhibits the expression and activity of NRPs, GmNAC81, and VPE and confer drought tolerance (Valente et al., 2009; Reis et al., 2011; Carvalho et al., 2014a). These previous results raised the hypothesis that mechanisms underlying drought tolerance may be associated with leaf longevity. To further examine this hypothesis, the *GmNAC81*-overexpressing lines, GmNAC81–1 and GmNAC81–3 at the developmental stage V3, were exposed to a progressive reduction of irrigation for 20 days. Under these dehydration conditions, BR16 and the transgenic lines GmNAC81–1 and GmNAC81–3 maintained similar water potential until the 12th day of water deprivation (Figure 3A). After 12 days, however, the leaf water potential decreased to a different extent in BR16 and the transgenic lines, resulting in accelerated leaf wilting in transgenic lines (Figure 3B). After 20 days under progressive dehydration, the leaf water potential of the stressed GmNAC81–3 line dropped to a maximum stress of −2.0 MPa, whereas the stressed GmNAC81–1 needed an additional day to reach this maximum stress. BR16 leaf water potential reached −2.0 MPa after 23 days of exposition to the restricted water regime. Under progressive dehydration, a clear difference in RWC between BR16 and transgenic (GmNAC81–1 and GmNAC81–3) leaves was observed at 17 days and persisted until 19 days after the treatment (Figure 3C). Collectively, these results indicate that, under progressive drought, *GmNAC81* overexpression enhances sensitivity to dehydration.

Under normal growth conditions, the photosynthetic rate (A) of transgenic lines was superior to that of BR16 (Figure 3D), which may explain the accelerated development of GmNAC81–1 and GmNAC81–3 lines (Pimenta et al., 2016). Accordingly, *GmNAC81* overexpression induced the expression of photosynthetic apparatus components (Supplementary Figure S5). We also showed that the precocious senescence of transgenic lines did not cause yield loss, which may be explained by the higher photosynthetic rate of the GmNAC81–1 and GmNAC81–3 lines. However, during progressive water deficit, the photosynthetic rate decline was more pronounced in the GmNAC81–1 and GmNAC81–3 lines compared to BR16, dropping to zero at 17 days in the transgenic lines but not until 20 days in BR16 (Figure 3D). The declines in stomatal conductance (gs) and transpiration rate (E) paralleled that of the photosynthetic rate (Supplementary Figures S6A,B). Therefore, under drought, the alterations in photosynthesis and transpiration rates may have been a direct result of stomatal closure, which occurred more rapidly in the transgenic lines; therefore, the mechanism for GmNAC81-mediated enhanced sensitivity to dehydration may not be associated with a failure in drought-induced stomatal closure.

Despite continuous stomatal closure during the progressive water deficit, the Ci/Ca ratio was higher in the *GmNAC81*-overexpressing lines than in the wild-type, indicating less CO_2_ fixation in the transgenic lines (Figure 3E). Only at 20 days of stress, the Ci/Ca ratio of BR16 was similar to that of the transgenic lines. During progressive water deprivation, the carboxylation efficiency (A/Ci ratio) was reduced more drastically in *GmNAC81*-overexpressing lines than in BR16 (Supplementary Figure S6C). Collectively, these results suggest that the more accentuated reduction in the CO_2_ assimilation rate in the *GmNAC081*-overexpressing lines during leaf dehydration might have been caused by biochemical damage of the photosynthetic apparatus.

The instantaneous water-use efficiency (A/E) progressively declined during the water deficit in both BR16 and transgenic lines, although to a different extent (Supplementary Figure S6D). A/E declined more rapidly and accentually in *GmNAC81*-overexpressing lines than in BR16, which maintained a superior A/E during all stress periods. The intrinsic water-use efficiency (A/gs) displayed the same trend as A/E (Supplementary Figure S6E). The photosynthetic activity of the soybean lines was also evaluated by measuring the maximum photochemical efficiency of PSII (Fv/Fm) (Figure 3F) and the electron transport rate (ETR) (Supplementary Figure S6F). Fv/Fm and ETR progressively declined during the water deficit regime, although significantly more in stressed GmNAC81–1 and GmNAC81–3 leaves than in stressed BR16 leaves. These results reflect a higher degree of drought-induced stress in the transgenic lines.

**FIGURE 4:**
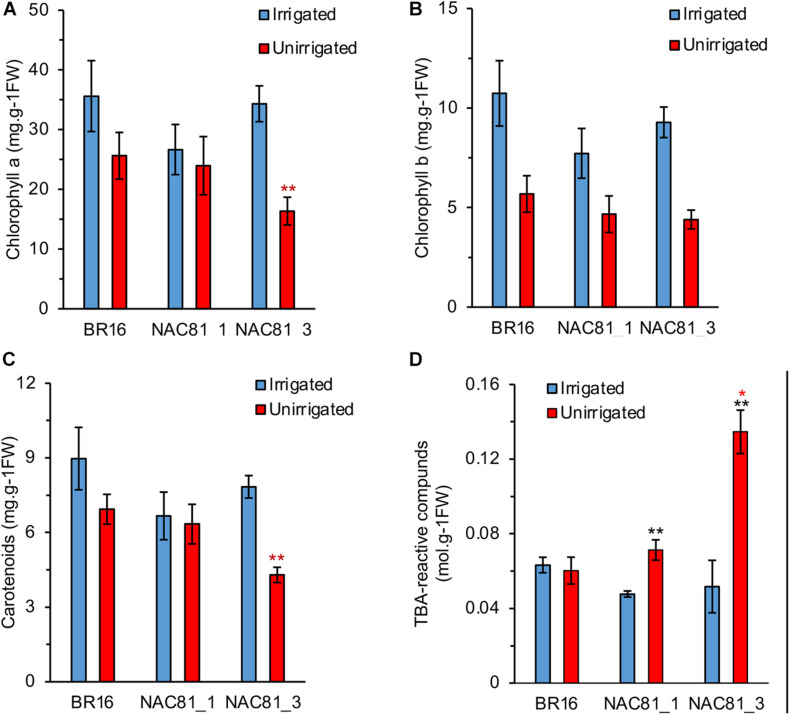
Pigment contents in drought-stressed soybeans plants. **(A)** Chlorophyll *a*. **(B)** Chlorophyll *b*. **(C)** Carotenoids. **(D)** Thiobarbituric acid-reactive compounds. The bars represent the standard error (*n* = 4). Significant results are indicated by *P < 0.05, **P < 0.01. The colored asterisks indicate the significantly different GmNAC81 line compared to BR16. The black asterisks indicate significant difference between treatments of same genotype.

Consistent with the enhanced drought susceptibility phenotype of GmNAC81–3, photosynthetic pigments, including chlorophyll *a*, chlorophyll *b*, and carotenoids, were more affected by water deficit in the GmNAC81–3 leaves than in GmNAC81–1 followed by BR16 (Figures 4A–C). Likewise, the extent of lipid peroxidation measured by the level of TBA-reactive compounds was higher in stressed GmNAC81–3 leaves than in stressed GmNAC81–1 and BR16 leaves (Figure 4D). These results correlated with the expression level of the GmNAC81, leaf gas-exchange data, and chlorophyll-fluorescence parameters. Furthermore, they confirmed that GmNAC81 is a negative modulator of drought tolerance.

As GmNAC81 is a downstream component of the multiple stress-induced DCD/NRP-mediated cell death response, the primary hypothesis was that GmNAC81 promotes susceptibility to drought due to its capacity to accelerate cell death induced by multiple stresses. We showed that GmNAC81 targets senescence-associated genes and accelerates senescence. These senescence-associated GmNAC81-regulated genes were common to the contrast GmNAC81_20DAG vs. BR16_20DAG and BR1_80DAG vs. BR16_20DAG. To examine whether GmNAC81 would also be directly involved in drought tolerance by targeting typical drought-responsive genes, we analyzed differentially expressed genes exclusively present in the contrast GmNAC81_20DAG vs. BR16_20DAG (Supplementary Table S5). Under normal growth conditions, the *GmNAC81* overexpression predominantly promoted the downregulation of drought-responsive genes. Among GmNAC81-repressed drought-responsive genes, we found major players in typical drought response. These include *PDR12* (AtPDR12)/ABCG40 (pleiotropic drug resistance transporter), which encodes a plasma membrane transporter that promotes ABA uptake (Kang et al., 2010), genes encoding aquaporins involved in water transport regulation in plant cells, such as PIP2A plasma membrane intrinsic protein 2A and TMP-C, plasma membrane intrinsic protein 1. Furthermore, GmNAC81 downregulated *CBF4*, a member of the DREB A-1 subfamily of TFs belonging to the ERF/AP2 family (Haake et al., 2002; Guttikonda et al., 2014). These results suggest that the drought sensitivity phenotype of *GmNAC81*-overexpressing lines may be associated not only with the GmNAC81 positive regulation of cell death and leaf senescence but also with the capacity of GmNAC81 to repress essential genes involved in the assembly of a drought-tolerant response.

## Discussion

GmNAC81 is a downstream component of the multiple stress- and natural leaf senescence-induced DCD/NRP-mediated cell death signaling. Here, we further confirmed the essential role of GmNAC81 as a positive regulator of developmentally programmed leaf senescence and uncovered a GmNAC81 negative role in drought response. Indeed, *GmNAC81* overexpression in the early vegetative stage accelerated leaf senescence and increased sensitivity to drought. Our data confirmed that the GmNAC81/VPE regulatory circuit accounts at least in part for the enhanced drought sensitivity of plants via induced PCD but also showed that GmNAC81 might act directly to modulate negatively typical drought response.

The genome-wide identification of the transcriptional activity of GmNAC81 revealed that the underlying mechanism for GmNAC81-mediated senescence is not only associated with the caspase 1-like VPE function, but also with upregulation of functionally characterized senescence-associated genes. Among the differentially expressed genes mediated by *GmNAC81* overexpression in the early vegetative stage (20 DAG), 25.3% upregulated and 42.7% downregulated genes were common to the transcriptional landscape of natural leaf senescence. In addition to previously described GmNAC81 targets, we found an overrepresentation of differentially expressed genes under biological categories related to cell death, leaf senescence, JA, and SA signaling. GmNAC81-regulated expression of six genes, *JMT*, *MLO3*, *NRT1.5*, *KIN10*, *KIT1*, and *SPI*, which function in leaf senescence, was further validated by RT-qPCR. These senescence-associated genes harbor GmNAC81 DNA binding sites [TGTG(TGC)] on their promoter, substantiating the argument that they may be direct targets for GmNAC81 control.

GmNAC81 also functions as a transcriptional repressor (Mendes et al., 2013), contributing to its positive function in leaf senescence. Approximately 43% of downregulated genes by GmNAC81 overexpression at 20 DAG were also downregulated by natural leaf senescence. PCD not only requires the activation of proteases, as pro-apoptotic factors but also the coordinate suppression of protease inhibitors to modulate the progress of cell death. As protease inhibitors and negative modulators of cell death during plant development and senescence, *KIT1* (Li et al., 2008) and *STI* (Clemente et al., 2019) were highly repressed by the *GmNAC81* overexpression at 20 DAG. Therefore, GmNAC81 not only directly targets VPE, as previously characterized (Mendes et al., 2013; Carvalho et al., 2014b), but also induces functionally described SAGs and suppresses inhibitors of leaf senescence, as potential direct targets.

In addition to accelerating leaf senescence, we provided several lines of evidence that GmNAC81 enhances sensitivity to water dehydration. First, under progressive water deficit, the GmNAC81-overexpressing lines, GmNAC81–1 and GmNAC81–3, maintained a lower leaf Ψw and RWC than the control, BR16. Second, progressive drought damaged the photosynthetic apparatus of the transgenic lines more rapidly than the control BR16. Accordingly, the photosynthesis rate, transpiration rate, and stomatal conductance declines were more pronounced in the transgenic lines, which displayed higher Ci/Ca and lower Fv/Fm ratios than BR16. Third, drought-induced pigment loss, including chlorophyll and carotenoids, and lipid peroxidation were higher in transgenic lines than in BR16, confirming that drought-induced cell death was enhanced in transgenic lines, which was consistent with the *GmNAC81*-induced transcriptome. These results also confirmed the hypothesis that modulation of the NRP-mediated cell death signaling is linked to drought response modulation. While ectopic expression of a negative modulator of this cell death signaling confers drought tolerance (Valente et al., 2009; Reis et al., 2011; Carvalho et al., 2014a), we showed here that overexpression of the positive regulator *GmNAC81* enhanced drought sensitivity.

Our results also indicated that cell death and other typical drought tolerance mechanisms might operate under the negative control of GmNAC81. Accordingly, drought-responsive genes were overrepresented in the set of genes specifically down-regulated by *GmNAC81* overexpression but not by leaf senescence. This set of genes uncouples GmNAC81-mediated leaf senescence from GmNAC81-mediated drought response. Among the GmNAC81 specifically down-regulated genes, we found classical TFs involved in drought response, including *CBF4*, a member of the DREB A-1 subfamily of the ERF/AP2 family (Haake et al., 2002; Guttikonda et al., 2014). CBF4 is a critical regulator of gene expression during drought adaptation and is induced by drought in an abscisic acid (ABA)-dependent manner. The ATP-binding cassette (ABC) transporter pleiotropic drug resistance transporter *PDR12* (AtPDR12)/ABCG40, a plasma membrane ABA uptake transporter, was also repressed in *GmNAC81*-overexpressing V3 leaves but not during the onset of leaf senescence (R6 leaves). Inactivation of *PDR12* in tobacco cells impairs ABA uptake and delays ABA-responsive genes (Kang et al., 2010). The Zinc finger protein *ZAT10* was found to be specifically downregulated by *GmNAC81* overexpression in V3 leaves. ZAT10 functions as both transcriptional activator and repressor involved in abiotic stress responses (Mittler et al., 2006); it controls the expression of the stress-responsive genes *DREB1A* and *LTI78*. Likewise, the regulators of ABA signaling, *AtHD2C* and *OST1*, were specifically downregulated in *GmNAC81*-overexpressing V3 leaves. As a functional drought-responsive gene, *AtHD2C* overexpression in Arabidopsis affects the expression of several ABA-responsive genes and enhances tolerance to salt and drought stresses (Sridha and Wu, 2006). OST1 is an ABA-activated protein kinase, which regulates stomatal closure by ABA but does not affect stomatal regulation by light and CO2, implicating OST1 specifically in ABA signaling (Mustilli et al., 2002). Collectively, these data suggest that GmNAC81 directly modulates drought tolerance by controlling critical regulators of ABA signaling. These results implicate the GmNAC81 regulatory function as a convergent link between stress-induced cell death signals and drought responses.

## Data Availability Statement

The datasets generated for this study can be found in the online repositories. The names of the repository/repositories and accession number(s) can be found in the article/Supplementary Material.

## Author Contributions

DF performed most of the experiments, analyzed the data, and wrote the first draft of the manuscript. OF performed the RT-qPCR. MP designed and assisted the drought stress experiments. HC assisted the drought stress experiments. JM and PC performed the RT-qPCR and carried-out RNA-seq. OB performed the bioinformatic and RNA-seq analysis. IQ performed the RT-qPCR of transgenic lines. PR designed the experiments. EF designed the experiments, analyzed the data, and wrote the manuscript. All authors contributed to the article and approved the submitted version.

## Conflict of Interest

The authors declare that the research was conducted in the absence of any commercial or financial relationships that could be construed as a potential conflict of interest.

## Footnotes

1 https://www.ncbi.nlm.nih.gov/geo/query/acc.cgi?acc=GSE159910

## Supplementary Material

Supplementary Figure 1 DAB staining of leaves at 80 DG and yield components of GmNAC81–1, GmNAC81–3, and BR16 plants.

Supplementary Figure 2 GmNAC81-mediated variation in the expression of senescence-related genes.

Supplementary Figure 3 Leaf senescence induces the expression of DCD/NRP-mediated programmed cell death signaling pathway.

Supplementary Figure 4 Phylogenetic tree of the SNAC-A subfamily (ATAF) of the NAC family.

Supplementary Figure 5 GmNAC81-mediated differential expression of the photosynthetic apparatus components [GmNAC81 (20DAG) × BR16 (20DAG)].

Supplementary Figure 6 Physiological parameters of gas exchange during progressive drought stress.

Supplementary Table 1 Enrichment analysis of genes differentially expressed [35S:GmNAC81 (20DAG)–BR16 (20 DAG)] related to senescence and the respective GO category.

Supplementary Table 2 Functional GO-based categorization of senescence related genes, differentially expressed in the contrast GmNAC81 (20 DAG)–BR16 (20 DAG).

Supplementary Table 3 Examples of genes differentially expressed shared by [35S:GmNAC81 (20DAG)–BR16 (20DAG)] vs. [35S:GmNAC81 (80 DAG)–BR16 (80DAG)].

Supplementary Table 4 Candidate targets of GmNAC81 harboring the binding site TGTG[T/G/C] in 5’ flanking sequences.

Supplementary Table 5 Genes differentially expressed involved in drought stress between 35S: GmNAC81 and BR16 plants at 20 DAG.

Supplementary Table 6 List of oligonucleotides used in the qRT-PCR assays.

Supplementary Methods Detailed description of Materials and Methods.
