# Supplementary material for: GmNAC81 Inversely Modulates Leaf Senescence and Drought Tolerance
Source: Front Genet. 2020 Nov 24;11:601876. doi: 10.3389/fgene.2020.601876 (PMC7732657; doi:10.3389/fgene.2020.601876)
Supplement: Supplementary Figure 1 — DAB staining of leaves at 80 DG and yield components of GmNAC81–1, GmNAC81–3, and BR16 plants. [file Data_Sheet_1.docx]

**Supplementary Figure S1**. DAB staining of leaves at 80 DAG and yield components of GmNAC81-1, GmNAC81-3 and BR16 plants. **(A)** Hydrogen peroxide accumulation in 80 DAG soybean leaves stained with DAB. **(B)** Yield components of soybean plants in the first growing season of experiments: number of pods per plant, number of seeds per pod and the weight of 1000 grains. **(C)** Yield components of soybean plants in the second growing season: number of pods per plant, number of seeds per pod and the weight of 1000 grains. Different letters indicate significantly different means among the genotypes (Tukey test *P* < 0.05).

**Supplementary Figure S2**. GmNAC81-mediated variation in the expression of senescence-related genes. **(A)** Hierarchical grouping of expression data of soybean genotype in different DAGs, using the WARD method and by correlation distance. **(B)** Genes upregulated in the contrasts [GmNAC81 (20DAG) -BR16(20DAG)] x [BR16(80DAG) – BR16(20DAG)]. **(C)** Genes downregulated in the contrasts [GmNAC81 (20DAG) -BR16(20DAG)] x [BR16(80DAG) – BR16(20DAG)].

**Supplementary Figure S3**. Leaf senescence induces the expression of DCD/NRP-mediated programmed cell death signaling pathway. **(A)** Differential expression of the signaling pathway components induced by natural senescence [BR16 (80DAG) – BR16 (20DAG)]. **(B)** Differential expression of the signaling pathway components induced by natural senescence in plants overexpressing GmNAC81 [GmNAC81 (80DAG) – GmNAC81 (20DAG)]. **(C)** Differential expression of the signaling pathway components induced by GmNAC081 expression at 20 DAG [GmNAC81 (20DAG) - BR16 (20DAG)]. The red indicates the genes downregulated and the green genes upregulated. **(D)** GmNAC81 targeted genes differentially expressed in the contrast GmNAC81_20DAG(V3) vs BR16_20DAG(V3).

**Supplementary Figure S4**. Phylogenetic tree of the SNAC-A subfamily (ATAF) of the NAC family. The amino acid sequences of similar proteins to GmNAC30 were obtained from the TAIR (http://arabidopsis.org/) or Phytozome v12.1 database and aligned using the MUSCLE program. The phylogenetic tree was built using Bayesian inference performed with MrBayes v3.2.2 with the mixed amino acid substitution model (Jones). The analyzes were carried out with 10,000,000 generations and excluding the first 2,500,000 generations as burn-in. The soybean NACs genes are represented in blue and Arabidopsis genes in red. The background colors demarcate clades formed by counterparts to GmNAC30. The external lines in red and blue demarcate the subgroup SNAC-A and SNAC-B (out-group), respectively. The species represented are *Arabidopsis thaliana*, *Solanum lycopercicum*, *Oryza sativa*, *Zea mays*, *Phaseolus vulgaris*, *Vitis vinífera*, *Gossypium raimondii*, *Eucalyptus grandis*, *Sorghum bicolor*, *Nicotiana benthamiana* and *Glycine max*.

**Supplementary Figure S5**. GmNAC081-mediated differential expression of the photosynthetic apparatus components, [GmNAC081 (20DAG) x BR16 (20DAG)]. Green indicates genes upregulated and red, genes downregulated.

**Supplementary Figure S6**. Physiological parameters of gas exchange during progressive drought stress. **(A)** Transpiratory ratio, **(B)** Stomatal conductance, **(C)** Carboxylation efficiency **(D)** Instantaneous water-use efficiency, and **(E)** Intrinsic water-use efficiency. Fluorescence physiological parameters during progressive drought stress **(F)** Electron transport rate. The bars represent the standard error (n = 4). Significant results are indicated by ∗*P* < 0.05, ∗∗*P* < 0.01. The colored asterisks match to the GmNAC81 line compared to BR16. NI indicated not irrigated.
