# Supplementary material for: GmNAC81 Inversely Modulates Leaf Senescence and Drought Tolerance
Source: Front Genet. 2020 Nov 24;11:601876. doi: 10.3389/fgene.2020.601876 (PMC7732657; doi:10.3389/fgene.2020.601876)
Supplement: Supplementary Table 1 — Enrichment analysis of genes differentially expressed [35S:GmNAC81 (20DAG)–BR16 (20 DAG)] related to senescence and the respective GO category. [file Data_Sheet_2.docx]

Table S1 - Enrichment analysis of genes differentially expressed [35S:GmNAC81 (20DAG) - BR16 (20 DAG)] related to senescence and the respective GO category.

| GO | Number found | Number in the group | *P-value* FDR |
| --- | --- | --- | --- |
| GO:0006624 vacuolar protein processing | 4 | 14 | 0.098 |
| GO:0007568 aging | 11 | 93 | 0.000 |
| GO:0007623 Circadian rhythm | 56 | 151 | 0.102 |
| GO:0008219 cell death | 29 | 109 | 0.000 |
| GO:0009611 response to wounding | 123 | 445 | 0.000 |
| GO:0009723 response to ethylene | 62 | 248 | 0.000 |
| GO:0009751 response to salicylic acid | 56 | 190 | 0.000 |
| GO:0009835 fruit ripening | 7 | 35 | 0.002 |
| GO:0010150 leaf senescence | 62 | 250 | 0.000 |
| GO:0012501 programmed cell death | 13 | 46 | 0.020 |
| GO:1900055 regulation of leaf senescence | 2 | 4 | 0.444 |
| GO:1900056 negative regulation of leaf senescence | 6 | 33 | 0.001 |
| GO:1900057 positive regulation of leaf senescence | 3 | 6 | 0.506 |

Table S2 - Functional GO-based categorization of senescence related genes, differentially expressed in the contrast GmNAC081 (20 DAG) – BR16 (20 DAG).

| **GO:0006624 vacuolar protein processing** | | | | |
| --- | --- | --- | --- | --- |
| **ID** | | **log_2_FC** | **padj** | **Description** |
| Glyma.12G129300 | | -0.61 | 0.002 | AT2G42700.1 |
| Glyma.05G055700 | | 0.94 | 0.002 | BETAVPE beta vacuolar processing enzyme |
| Glyma.14G092800 | | 2.31 | 0.000 | GAMMAVPE gamma vacuolar processing enzyme |
| Glyma.17G230700 | | 3.25 | 0.000 | GAMMAVPE gamma vacuolar processing enzyme |
| **GO:0007568 aging** | | | | |
| **ID** | | **log_2_FC** | **padj** | **Description** |
| Glyma.20G036100 | | -2.39 | 0.012 | RNS1 ribonuclease 1 |
| Glyma.04G147100 | | -1.17 | 0.022 | TET7 tetraspanin7 |
| Glyma.08G029100 | | -0.66 | 0.006 | TET2 tetraspanin2 |
| Glyma.13G091000 | | -0.52 | 0.029 | TET8 tetraspanin8 |
| Glyma.07G059300 | | 0.48 | 0.016 | RNS2 ribonuclease 2 |
| Glyma.19G013200 | | 0.58 | 0.014 | SAG101 senescence-associated gene 101 |
| Glyma.10G112000 | | 0.63 | 0.031 | CCH copper chaperone |
| Glyma.13G210800 | | 0.69 | 0.049 | GS2 glutamine synthetase 2 |
| Glyma.15G102000 | | 0.91 | 0.002 | GS2 glutamine synthetase 2 |
| Glyma.04G190200 | | 1.03 | 0.000 | PIMT1 protein-l-isoaspartate methyltransferase 1 |
| Glyma.12G109200 | | 1.64 | 0.012 | GS2 glutamine synthetase 2 |
| **GO:0007623 Circadian rhythm** | | | | |
| **ID** | | **log_2_FC** | **padj** | **Description** |
| Glyma.17G172400 | | -2.75 | 0.000 | SPT basic helix-loop-helix (bHLH) DNA-binding superfamily protein |
| Glyma.06G114900 | | -2.11 | 0.000 | ATRR4 response regulator 9 |
| Glyma.13G244200 | | -1.90 | 0.022 | SRF3 STRUBBELIG-receptor family 3 |
| Glyma.17G126400 | | -1.82 | 0.000 | BT2 BTB and TAZ domain protein 2 |
| Glyma.11G054600 | | -1.72 | 0.000 | SPT basic helix-loop-helix (bHLH) DNA-binding superfamily protein |
| Glyma.13G184200 | | -1.64 | 0.039 | SRF3 STRUBBELIG-receptor family 3 |
| Glyma.05G239400 | | -1.57 | 0.005 | ADO3,FKF1 flavin-binding, kelch repeat, f box 1 |
| Glyma.11G155100 | | -1.44 | 0.006 | ATRR4 response regulator 9 |
| Glyma.19G110200 | | -1.29 | 0.002 | GATA9 GATA transcription factor 9 |
| Glyma.12G074100 | | -1.22 | 0.000 | JK224,NPH1,PHOT1,RPT1 phototropin 1 |
| Glyma.17G093900 | | -1.20 | 0.000 | RR5 response regulator 5 |
| Glyma.05G033000 | | -1.10 | 0.002 | RR5 response regulator 5 |
| Glyma.06G296800 | | -1.10 | 0.028 | SRF3 STRUBBELIG-receptor family 3 |
| Glyma.17G037400 | | -1.05 | 0.002 | Plant neutral invertase family protein |
| Glyma.05G067100 | | -0.87 | 0.000 | CKB1 casein kinase II beta chain 1 |
| Glyma.15G074000 | | -0.77 | 0.000 | GSL1 glucan synthase-like 1 |
| Glyma.20G060400 | | -0.75 | 0.006 | COL9 CONSTANS-like 9 |
| Glyma.02G296300 | | -0.68 | 0.007 | FIM1 fimbrin 1 |
| Glyma.14G094800 | | -0.67 | 0.024 | GATA22 cytokinin-responsive gata factor 1 |
| Glyma.11G117600 | | -0.66 | 0.000 | GRP7 cold, circadian rhythm, and rna binding 2 |
| Glyma.12G043000 | | -0.64 | 0.001 | CCR1,GR-RBP8,GRP8 cold, circadian rhythm, and RNA binding 1 |
| Glyma.11G230200 | | -0.62 | 0.023 | ATNFXL2 sequence-specific DNA binding transcription factors; zinc ion binding;sequence-specific DNA binding transcription factors |
| Glyma.11G054800 | | -0.59 | 0.025 | Protein kinase superfamily protein |
| Glyma.01G187300 | | -0.54 | 0.022 | Protein kinase superfamily protein |
| Glyma.19G242900 | | -0.43 | 0.027 | WNK1,ZIK4 with no lysine (K) kinase 1 |
| Glyma.04G096200 | | -0.39 | 0.013 | TIFY1,ZIM GATA-type zinc finger protein with TIFY domain |
| Glyma.13G135900 | | -0.37 | 0.000 | PRR7 pseudo-response regulator 7 |
| Glyma.01G200800 | | 0.32 | 0.045 | PRR2 CheY-like two-component responsive regulator family protein |
| Glyma.15G162300 | | 0.33 | 0.005 | ZTL Galactose oxidase/kelch repeat superfamily protein |
| Glyma.06G314300 | | 0.37 | 0.000 | FHY3 far-red elongated hypocotyls 3 |
| Glyma.05G157200 | | 0.45 | 0.018 | MPK7 MAP kinase 7 |
| Glyma.16G018000 | | 0.46 | 0.035 | PRR5 pseudo-response regulator 5 |
| Glyma.08G218100 | | 0.47 | 0.007 | IRX4 cinnamoyl coa reductase 1 |
| Glyma.10G248400 | | 0.49 | 0.010 | WNK1,ZIK4 with no lysine (K) kinase 1 |
| Glyma.04G124300 | | 0.50 | 0.000 | FHY3 far-red elongated hypocotyls 3 |
| Glyma.17G152800 | | 0.54 | 0.000 | PRR2 CheY-like two-component responsive regulator family protein |
| Glyma.19G260400 | | 0.55 | 0.000 | PRR5 pseudo-response regulator 5 |
| Glyma.19G227700 | | 0.55 | 0.041 | CSP41B chloroplast RNA binding |
| Glyma.09G056100 | | 0.58 | 0.000 | ZTL Galactose oxidase/kelch repeat superfamily protein |
| Glyma.07G049400 | | 0.61 | 0.001 | PRR5 pseudo-response regulator 5 |
| Glyma.20G228000 | | 0.62 | 0.006 | WNK1,ZIK4 with no lysine (K) kinase 1 |
| Glyma.10G160400 | | 0.65 | 0.000 | WNK1,ZIK4 with no lysine (K) kinase 1 |
| Glyma.03G261300 | | 0.66 | 0.000 | PRR5 pseudo-response regulator 5 |
| Glyma.02G100000 | | 0.74 | 0.037 | STN7 STT7 homolog STN7 |
| Glyma.03G230000 | | 0.78 | 0.019 | CSP41B chloroplast RNA binding |
| Glyma.04G012700 | | 0.79 | 0.000 | GRP7 cold, circadian rhythm, and rna binding 2 |
| Glyma.06G136600 | | 0.80 | 0.022 | PRR5 pseudo-response regulator 5 |
| Glyma.06G012600 | | 0.82 | 0.000 | GRP7 cold, circadian rhythm, and rna binding 2 |
| Glyma.01G087900 | | 0.85 | 0.001 | STN7 STT7 homolog STN7 |
| Glyma.09G062800 | | 0.88 | 0.000 | GNC GATA type zinc finger transcription factor family protein |
| Glyma.08G274400 | | 0.90 | 0.000 | STM serine transhydroxymethyltransferase 1 |
| Glyma.18G150000 | | 0.96 | 0.000 | STM serine transhydroxymethyltransferase 1 |
| Glyma.15G043600 | | 0.98 | 0.000 | JK224,NPH1,PHOT1,RPT1 phototropin 1 |
| Glyma.17G055200 | | 0.99 | 0.004 | GNC GATA type zinc finger transcription factor family protein |
| Glyma.02G073900 | | 1.19 | 0.001 | GATA transcription factor 4 |
| Glyma.04G190900 | | 1.64 | 0.013 | BT2 BTB and TAZ domain protein 2 |
| **GO:0008219 cell death** | | | | |
| **ID** | **log_2_FC** | | **padj** | **Description** |
| Glyma.14G081300 | -4.08 | | 0.000 | PLP2 phospholipase A 2A |
| Glyma.16G080100 | -1.26 | | 0.000 | MLO1 Seven transmembrane MLO family protein |
| Glyma.14G098000 | -1.25 | | 0.003 | GPA1 G protein alpha subunit 1 |
| Glyma.04G056600 | -1.13 | | 0.015 | GPA1 G protein alpha subunit 1 |
| Glyma.16G111900 | -1.12 | | 0.001 | MLO8 Seven transmembrane MLO family protein |
| Glyma.20G178800 | -0.86 | | 0.035 | MLO6 Seven transmembrane MLO family protein |
| Glyma.09G010000 | -0.73 | | 0.000 | Fumarylacetoacetase, putative |
| Glyma.09G196800 | -0.66 | | 0.027 | MLO8 Seven transmembrane MLO family protein |
| Glyma.12G159600 | -0.27 | | 0.045 | RNA-binding (RRM/RBD/RNP motifs) family protein |
| Glyma.14G040800 | -0.25 | | 0.027 | RNA-binding (RRM/RBD/RNP motifs) family protein |
| Glyma.13G145100 | 0.28 | | 0.004 | PIR2 NRAMP metal ion transporter family protein |
| Glyma.11G087500 | 0.41 | | 0.022 | MLO8 Seven transmembrane MLO family protein |
| Glyma.03G118800 | 0.42 | | 0.019 | RNA-binding (RRM/RBD/RNP motifs) family protein |
| Glyma.01G157500 | 0.42 | | 0.019 | MLO8 Seven transmembrane MLO family protein |
| Glyma.20G229500 | 0.44 | | 0.000 | EDR1 Protein kinase superfamily protein |
| Glyma.07G205500 | 0.58 | | 0.000 | RNA-binding (RRM/RBD/RNP motifs) family protein |
| Glyma.12G082100 | 0.59 | | 0.008 | PAO Pheophorbide a oxygenase family protein with Rieske [2Fe-2S] domain |
| Glyma.08G074500 | 0.59 | | 0.005 | SERK3 BRI1-associated receptor kinase |
| Glyma.17G261700 | 0.59 | | 0.000 | CAT2 catalase 2 |
| Glyma.03G168700 | 0.67 | | 0.000 | PAR2 GroES-like zinc-binding dehydrogenase family protein |
| Glyma.06G017900 | 0.69 | | 0.001 | CAT2 catalase 2 |
| Glyma.04G002400 | 0.76 | | 0.000 | MLO1 Seven transmembrane MLO family protein |
| Glyma.11G192300 | 0.82 | | 0.000 | PAO Pheophorbide a oxygenase family protein with Rieske [2Fe-2S] domain |
| Glyma.20G125200 | 0.89 | | 0.016 | RPI2 ribose-5-phosphate isomerase 2 |
| Glyma.04G017500 | 0.93 | | 0.000 | CAT2 catalase 2 |
| Glyma.10G212300 | 1.24 | | 0.000 | MLO6 Seven transmembrane MLO family protein |
| Glyma.12G070600 | 1.95 | | 0.000 | MLO6 Seven transmembrane MLO family protein |
| Glyma.03G179200 | 3.11 | | 0.000 | MLO3 Seven transmembrane MLO family protein |
| Glyma.19G179900 | 4.36 | | 0.000 | MLO3 Seven transmembrane MLO family protein |
| **GO:0009611 response to wounding** | | | | |
| **ID** | **log_2_FC** | | **padj** | **Description** |
| Glyma.08G235400 | -6.88 | | 0.000 | ATKTI1,KTI1 kunitz trypsin inhibitor 1 |
| Glyma.06G220800 | -6.44 | | 0.000 | ATBCB,BCB,SAG14 blue-copper-binding protein |
| Glyma.10G184600 | -5.95 | | 0.000 | Serine protease inhibitor, potato inhibitor I-type family protein |
| Glyma.07G034900 | -5.87 | | 0.000 | ATLOX1,LOX1 lipoxygenase 1 |
| Glyma.20G205800 | -5.77 | | 0.000 | Serine protease inhibitor, potato inhibitor I-type family protein |
| Glyma.10G184700 | -5.17 | | 0.000 | Serine protease inhibitor, potato inhibitor I-type family protein |
| Glyma.04G035000 | -4.65 | | 0.000 | AOS,CYP74A,DDE2 allene oxide synthase |
| Glyma.15G223900 | -4.34 | | 0.000 | ATOPR2,OPR2 12-oxophytodienoate reductase 2 |
| Glyma.08G342100 | -4.20 | | 0.002 | ATKTI1,KTI1 kunitz trypsin inhibitor 1 |
| Glyma.08G341700 | -3.36 | | 0.000 | ATKTI1,KTI1 kunitz trypsin inhibitor 1 |
| Glyma.08G341800 | -3.36 | | 0.001 | ATKTI1,KTI1 kunitz trypsin inhibitor 1 |
| Glyma.14G103100 | -3.34 | | 0.000 | ATWRKY40,WRKY40 WRKY DNA-binding protein 40 |
| Glyma.13G030300 | -3.04 | | 0.000 | ATLOX2,LOX2 lipoxygenase 2 |
| Glyma.08G189600 | -2.99 | | 0.000 | ATLOX1,LOX1 lipoxygenase 1 |
| Glyma.09G126200 | -2.81 | | 0.015 | ATCHIA,CHIA chitinase A |
| Glyma.14G102900 | -2.70 | | 0.001 | ATWRKY40,WRKY40 WRKY DNA-binding protein 40 |
| Glyma.03G160200 | -2.70 | | 0.021 | CYP94B1 cytochrome P450, family 94, subfamily B, polypeptide 1 |
| Glyma.08G341600 | -2.62 | | 0.000 | ATKTI1,KTI1 kunitz trypsin inhibitor 1 |
| Glyma.13G346700 | -2.56 | | 0.000 | ATCHITIV,ATEP3,CHIV,EP3 homolog of carrot EP3-3 chitinase |
| Glyma.08G342300 | -2.52 | | 0.031 | ATKTI1,KTI1 kunitz trypsin inhibitor 1 |
| Glyma.13G370100 | -2.50 | | 0.000 | ATWRKY40,WRKY40 WRKY DNA-binding protein 40 |
| Glyma.13G186200 | -2.42 | | 0.029 | ATOPR2,OPR2 12-oxophytodienoate reductase 2 |
| Glyma.20G036100 | -2.39 | | 0.012 | ATRNS1,RNS1 ribonuclease 1 |
| Glyma.08G003900 | -2.37 | | 0.014 | ATDGK2,DGK2 diacylglycerol kinase 2 |
| Glyma.01G228700 | -2.08 | | 0.005 | TT4 Chalcone and stilbene synthase family protein |
| Glyma.16G173000 | -2.03 | | 0.002 | ATCHIA,CHIA chitinase A |
| Glyma.08G109500 | -2.01 | | 0.046 | ATCHS,CHS,TT4 Chalcone and stilbene synthase family protein |
| Glyma.10G005300 | -1.88 | | 0.002 | ATPTR3,PTR3 peptide transporter 3 |
| Glyma.17G126400 | -1.82 | | 0.000 | ATBT2,BT2 BTB and TAZ domain protein 2 |
| Glyma.08G218600 | -1.66 | | 0.009 | ATWRKY40,WRKY40 WRKY DNA-binding protein 40 |
| Glyma.06G061900 | -1.63 | | 0.019 | WRKY40 WRKY DNA-binding protein 40 |
| Glyma.11G124500 | -1.55 | | 0.004 | ATCHITIV,ATEP3,CHIV,EP3 homolog of carrot EP3-3 chitinase |
| Glyma.11G197500 | -1.51 | | 0.016 | PLAT/LH2 domain-containing lipoxygenase family protein |
| Glyma.16G020200 | -1.51 | | 0.025 | CIPK9,PKS6,SnRK3.12 CBL-interacting protein kinase 9 |
| Glyma.20G054000 | -1.41 | | 0.000 | ATLOX2,LOX2 lipoxygenase 2 |
| Glyma.03G260200 | -1.39 | | 0.000 | CIPK9,PKS6,SnRK3.12 CBL-interacting protein kinase 9 |
| Glyma.13G347800 | -1.36 | | 0.043 | ATLOX1,LOX1 lipoxygenase 1 |
| Glyma.07G196800 | -1.34 | | 0.042 | LOX3 lipoxygenase 3 |
| Glyma.13G043500 | -1.30 | | 0.006 | ABCG11,ATWBC11,COF1,DSO,WBC11 white-brown complex homolog protein 11 |
| Glyma.08G018000 | -1.24 | | 0.024 | ACS6,ATACS6 1-aminocyclopropane-1-carboxylic acid (acc) synthase 6 |
| Glyma.15G026400 | -1.21 | | 0.033 | ATLOX1,LOX1 lipoxygenase 1 |
| Glyma.06G157400 | -1.17 | | 0.021 | ANAC002,ATAF1 NAC (No Apical Meristem) domain transcriptional  regulator superfamily protein |
| Glyma.04G007700 | -1.13 | | 0.000 | SPE2 arginine decarboxylase 2 |
| Glyma.07G059000 | -1.10 | | 0.000 | Pyridoxal phosphate (PLP)-dependent transferases superfamily protein |
| Glyma.13G239000 | -1.06 | | 0.004 | LOX3 lipoxygenase 3 |
| Glyma.18G118100 | -1.05 | | 0.000 | SQE1,XF1 FAD/NAD(P)-binding oxidoreductase family protein |
| Glyma.16G088000 | -0.99 | | 0.000 | ATFKBP62,FKBP62,ROF1 rotamase FKBP 1 |
| Glyma.19G009100 | -0.99 | | 0.010 | FDH formate dehydrogenase |
| Glyma.08G197300 | -0.98 | | 0.008 | IAR3,JR3 peptidase M20/M25/M40 family protein |
| Glyma.11G122700 | -0.94 | | 0.006 | AOS,CYP74A,DDE2 allene oxide synthase |
| Glyma.13G088100 | -0.90 | | 0.001 | RAP2.4 related to AP2 4 |
| Glyma.09G160000 | -0.90 | | 0.000 | ABCG11,ATWBC11,COF1,DSO,WBC11 white-brown complex homolog protein 11 |
| Glyma.14G171500 | -0.88 | | 0.013 | RAP2.4 related to AP2 4 |
| Glyma.03G086200 | -0.69 | | 0.013 | FKBP62,ROF1 rotamase FKBP 1 |
| Glyma.20G136300 | -0.67 | | 0.001 | Inositol 1,3,4-trisphosphate 5/6-kinase family protein |
| Glyma.11G035200 | -0.66 | | 0.000 | ACX1,ATACX1 acyl-CoA oxidase 1 |
| Glyma.09G070500 | -0.59 | | 0.000 | ATSSL4,SSL4 strictosidine synthase-like 4 |
| Glyma.09G044500 | -0.49 | | 0.006 | ATMRP4,EST3,MRP4 multidrug resistance-associated protein 4 |
| Glyma.20G077000 | -0.49 | | 0.000 | KAT2,PED1,PKT3 peroxisomal 3-ketoacyl-CoA thiolase 3 |
| Glyma.16G111700 | -0.47 | | 0.020 | ATGLR3.6,GLR3.6 glutamate receptor 3.6 |
| Glyma.06G016000 | -0.44 | | 0.044 | GLR3.3 glutamate receptor 3.3 |
| Glyma.01G096600 | -0.40 | | 0.046 | ZBF1 Basic helix-loop-helix (bHLH) DNA-binding family protein |
| Glyma.08G346700 | -0.36 | | 0.030 | ATFKBP62,FKBP62,ROF1 rotamase FKBP 1 |
| Glyma.01G202600 | 0.18 | | 0.027 | UPF1 RNA helicase, putative |
| Glyma.17G022300 | 0.19 | | 0.045 | CHR3,SYD P-loop containing nucleoside triphosphate hydrolases superfamily protein |
| Glyma.09G087900 | 0.22 | | 0.009 | Binding;RNA binding |
| Glyma.06G072200 | 0.31 | | 0.002 | LUG,RON2 LisH dimerisation motif;WD40/YVTN repeat-like-containing domain |
| Glyma.18G269500 | 0.32 | | 0.004 | HRT,RCY1,RPP8 Disease resistance protein (CC-NBS-LRR class) family |
| Glyma.15G194000 | 0.33 | | 0.020 | Binding;RNA binding |
| Glyma.13G352400 | 0.34 | | 0.024 | IAR3,JR3 peptidase M20/M25/M40 family protein |
| Glyma.02G297400 | 0.36 | | 0.023 | WRKY20 WRKY family transcription factor family protein |
| Glyma.11G012000 | 0.39 | | 0.009 | AT-RSH3,ATRSH3,RSH3 RELA/SPOT homolog 3 |
| Glyma.03G167900 | 0.40 | | 0.021 | Pyridoxal phosphate (PLP)-dependent transferases superfamily protein |
| Glyma.07G180100 | 0.40 | | 0.045 | KAT2,PED1,PKT3 peroxisomal 3-ketoacyl-CoA thiolase 3 |
| Glyma.13G084100 | 0.43 | | 0.005 | ARAKIN,ATMEKK1,MAPKKK8,MEKK1 MAPK/ERK kinase kinase1 |
| Glyma.08G271900 | 0.43 | | 0.004 | ATMYC2,JAI1,JIN1,MYC2,RD22BP1,ZBF1 Basic helix-loop-helix (bHLH) DNA-binding family protein |
| Glyma.18G171100 | 0.45 | | 0.000 | LUH LEUNIG_homolog |
| Glyma.06G089800 | 0.47 | | 0.002 | Inositol 1,3,4-trisphosphate 5/6-kinase family protein |
| Glyma.05G123200 | 0.48 | | 0.024 | ARAKIN,ATMEKK1,MAPKKK8,MEKK1 MAPK/ERK kinase kinase1 |
| Glyma.08G078200 | 0.51 | | 0.005 | ARAKIN,ATMEKK1,MAPKKK8,MEKK1 MAPK/ERK kinase kinase1 |
| Glyma.04G088000 | 0.52 | | 0.010 | Inositol 1,3,4-trisphosphate 5/6-kinase family protein |
| Glyma.03G216400 | 0.52 | | 0.027 | RSH1 RELA/SPOT homolog 1 |
| Glyma.19G227700 | 0.55 | | 0.041 | CRB,CSP41B chloroplast RNA binding |
| Glyma.07G057900 | 0.59 | | 0.003 | FIN219,JAR1 Auxin-responsive GH3 family protein |
| Glyma.08G354500 | 0.60 | | 0.000 | LUH LEUNIG_homolog |
| Glyma.12G099500 | 0.61 | | 0.004 | ATGLR3.4,GLR3.4,GLUR3 glutamate receptor 3.4 |
| Glyma.11G130200 | 0.62 | | 0.000 | ATLOX2,LOX2 lipoxygenase 2 |
| Glyma.09G191300 | 0.63 | | 0.015 | ATPERK1,PERK1 proline extensin-like receptor kinase 1 |
| Glyma.19G210700 | 0.63 | | 0.021 | ATMRP4,EST3,MRP4 multidrug resistance-associated protein 4 |
| Glyma.19G212900 | 0.63 | | 0.007 | AT-RSH1,ATRSH1,RSH1 RELA/SPOT homolog 1 |
| Glyma.04G249000 | 0.65 | | 0.001 | ANAC002,ATAF1 NAC (No Apical Meristem) domain transcriptional regulator superfamily protein |
| Glyma.14G152700 | 0.67 | | 0.000 | ANAC002,ATAF1 NAC (No Apical Meristem) domain transcriptional regulator superfamily protein |
| Glyma.11G087100 | 0.71 | | 0.000 | ATGLR3.6,GLR3.6 glutamate receptor 3.6 |
| Glyma.10G019000 | 0.71 | | 0.042 | ATMRP4,EST3,MRP4 multidrug resistance-associated protein 4 |
| Glyma.15G128000 | 0.72 | | 0.005 | Pyridoxal phosphate (PLP)-dependent transferases superfamily protein |
| Glyma.20G164900 | 0.73 | | 0.010 | ATCHIA,CHIA chitinase A |
| Glyma.03G230000 | 0.78 | | 0.019 | CSP41B chloroplast RNA binding |
| Glyma.16G026900 | 0.79 | | 0.001 | FIN219,JAR1 Auxin-responsive GH3 family protein |
| Glyma.01G235600 | 0.85 | | 0.011 | OPR2 12-oxophytodienoate reductase 2 |
| Glyma.12G054700 | 0.85 | | 0.000 | ATLOX2,LOX2 lipoxygenase 2 |
| Glyma.07G013300 | 0.86 | | 0.002 | IAR3,JR3 peptidase M20/M25/M40 family protein |
| Glyma.11G085100 | 0.91 | | 0.000 | RHD4 Phosphoinositide phosphatase family protein |
| Glyma.16G008700 | 0.92 | | 0.000 | LOX3 lipoxygenase 3 |
| Glyma.13G095600 | 0.93 | | 0.001 | 4CL2,AT4CL2 4-coumarate:CoA ligase 2 |
| Glyma.U025000 | 0.99 | | 0.004 | AtPSKR2,PSKR2 phytosylfokine-alpha receptor 2 |
| Glyma.06G305000 | 1.01 | | 0.000 | ATGLR3.4,GLR3.4,GLUR3 glutamate receptor 3.4 |
| Glyma.10G255000 | 1.04 | | 0.000 | Inositol 1,3,4-trisphosphate 5/6-kinase family protein |
| Glyma.20G206300 | 1.11 | | 0.000 | ATPTR3,PTR3 peptide transporter 3 |
| Glyma.10G183900 | 1.12 | | 0.000 | ATPTR3,PTR3 peptide transporter 3 |
| Glyma.14G223600 | 1.16 | | 0.000 | ATOPR1,OPR1 12-oxophytodienoate reductase 1 |
| Glyma.13G361200 | 1.22 | | 0.002 | AXR3,IAA17 AUX/IAA transcriptional regulator family protein |
| Glyma.11G239200 | 1.53 | | 0.001 | RAP2.4 related to AP2 4 |
| Glyma.11G130300 | 1.56 | | 0.000 | ATLOX2,LOX2 lipoxygenase 2 |
| Glyma.19G263300 | 1.56 | | 0.004 | LOX3 lipoxygenase 3 |
| Glyma.04G190900 | 1.64 | | 0.013 | ATBT2,BT2 BTB and TAZ domain protein 2 |
| Glyma.07G162900 | 1.67 | | 0.009 | AOS,CYP74A,DDE2 allene oxide synthase |
| Glyma.18G132000 | 1.69 | | 0.000 | IAR3,JR3 peptidase M20/M25/M40 family protein |
| Glyma.15G074300 | 1.70 | | 0.000 | PLAT/LH2 domain-containing lipoxygenase family protein |
| Glyma.10G153900 | 2.21 | | 0.000 | ATLOX1,LOX1 lipoxygenase 1 |
| Glyma.14G092800 | 2.31 | | 0.000 | GAMMA-VPE,GAMMAVPE gamma vacuolar processing enzyme |
| Glyma.01G193300 | 3.24 | | 0.000 | BT4 BTB and TAZ domain protein 4 |
| Glyma.17G230700 | 3.25 | | 0.000 | GAMMA-VPE,GAMMAVPE gamma vacuolar processing enzyme |
| Glyma.14G072300 | 3.48 | | 0.000 | JMT jasmonic acid carboxyl methyltransferase |
| **GO:0009723 response to ethylene** | | | | |
| **ID** | **log_2_FC** | | **padj** | **Description** |
| Glyma.07G033500 | -5.22 | | 0.000 | ABCG40,ATABCG40,ATPDR12,PDR12 pleiotropic drug resistance 12 |
| Glyma.15G012000 | -5.12 | | 0.000 | ABCG40,ATABCG40,ATPDR12,PDR12 pleiotropic drug resistance 12 |
| Glyma.03G247500 | -3.25 | | 0.000 | HEL,PR-4,PR4 pathogenesis-related 4 |
| Glyma.03G168000 | -2.61 | | 0.005 | ABCG40,ATABCG40,ATPDR12,PDR12 pleiotropic drug resistance 12 |
| Glyma.06G247100 | -1.89 | | 0.000 | PORA protochlorophyllide oxidoreductase A |
| Glyma.06G317500 | -1.78 | | 0.000 | ATPHB3,PHB3 prohibitin 3 |
| Glyma.07G130400 | -1.70 | | 0.000 | ARF7,BIP,IAA21,IAA23,IAA25,MSG1,NPH4,TIR5 Transcriptional factor B3 family protein / auxin-responsive factor AUX/IAA-related |
| Glyma.04G147000 | -1.66 | | 0.003 | EBF1,FBL6 EIN3-binding F box protein 1 |
| Glyma.19G245400 | -1.49 | | 0.000 | HEL,PR-4,PR4 pathogenesis-related 4 |
| Glyma.13G361900 | -1.45 | | 0.000 | ABCG40,ATABCG40,ATPDR12,PDR12 pleiotropic drug resistance 12 |
| Glyma.02G122000 | -1.39 | | 0.006 | FER Malectin/receptor-like protein kinase family protein |
| Glyma.08G018000 | -1.24 | | 0.024 | ACS6,ATACS6 1-aminocyclopropane-1-carboxylic acid (acc) synthase 6 |
| Glyma.05G021100 | -1.17 | | 0.042 | ATRBOH F,ATRBOHF,RBOH F,RBOHAP108,RBOHF respiratory burst oxidase protein F |
| Glyma.09G209900 | -1.12 | | 0.001 | XLG3 extra-large GTP-binding protein 3 |
| Glyma.08G018900 | -1.05 | | 0.025 | ATRBOH F,ATRBOHF,RBOH F,RBOHAP108,RBOHF respiratory burst oxidase protein F |
| Glyma.13G166200 | -0.96 | | 0.002 | EBF1,FBL6 EIN3-binding F box protein 1 |
| Glyma.09G072200 | -0.81 | | 0.007 | ARF7,BIP,IAA21,IAA23,IAA25,MSG1,NPH4,TIR5 Transcriptional factor B3 family protein / auxin-responsive factor AUX/IAA-related |
| Glyma.16G119000 | -0.72 | | 0.000 | SSL5,YLS2 Calcium-dependent phosphotriesterase superfamily protein |
| Glyma.01G002100 | -0.68 | | 0.001 | ARF7,BIP,IAA21,IAA23,IAA25,MSG1,NPH4,TIR5 Transcriptional factor B3 family protein / auxin-responsive factor AUX/IAA-related |
| Glyma.03G191000 | 0.25 | | 0.049 | AtCTR1,CTR1,SIS1 Protein kinase superfamily protein |
| Glyma.13G145100 | 0.28 | | 0.004 | ATEIN2,CKR1,EIN2,ERA3,ORE2,ORE3,PIR2 NRAMP metal ion transporter family protein |
| Glyma.09G002600 | 0.30 | | 0.030 | AtETR1,EIN1,ETR,ETR1 Signal transduction histidine kinase, hybrid-type, ethylene sensor |
| Glyma.14G031700 | 0.32 | | 0.028 | transducin family protein / WD-40 repeat family protein |
| Glyma.08G240800 | 0.37 | | 0.001 | WRKY4 WRKY DNA-binding protein 4 |
| Glyma.01G147800 | 0.42 | | 0.000 | EDR2 ENHANCED DISEASE RESISTANCE 2 |
| Glyma.03G041600 | 0.43 | | 0.001 | ATSCAR2,DIS3,ITB1,SCAR2,WAVE4 SCAR homolog 2 |
| Glyma.20G229500 | 0.44 | | 0.000 | ATEDR1,EDR1 Protein kinase superfamily protein |
| Glyma.14G116800 | 0.45 | | 0.024 | EBF1,FBL6 EIN3-binding F box protein 1 |
| Glyma.19G242400 | 0.45 | | 0.002 | AIN1,ATXRN4,EIN5,XRN4 exoribonuclease 4 |
| Glyma.15G158300 | 0.47 | | 0.006 | HMA7,RAN1 copper-exporting ATPase / responsive-to-antagonist 1 /copper-transporting ATPase (RAN1) |
| Glyma.02G085900 | 0.49 | | 0.006 | ARR2,RR2 response regulator 2 |
| Glyma.18G270700 | 0.51 | | 0.027 | FER Malectin/receptor-like protein kinase family protein |
| Glyma.18G270600 | 0.54 | | 0.003 | FER Malectin/receptor-like protein kinase family protein |
| Glyma.13G151100 | 0.55 | | 0.004 | AtCTR1,CTR1,SIS1 Protein kinase superfamily protein |
| Glyma.06G068400 | 0.56 | | 0.000 | EBF1,FBL6 EIN3-binding F box protein 1 |
| Glyma.05G106000 | 0.57 | | 0.003 | ATTBP3,ATTRB2,TBP3,TRB2 Homeodomain-like/winged-helix DNA-binding family protein |
| Glyma.14G218000 | 0.58 | | 0.000 | AtXRN3,XRN3 5\`-3\` exoribonuclease 3 |
| Glyma.07G243300 | 0.60 | | 0.005 | ARR2,RR2 response regulator 2 |
| Glyma.08G027800 | 0.60 | | 0.025 | ARF7,BIP,IAA21,IAA23,IAA25,MSG1,NPH4,TIR5 Transcriptional factor B3 family protein / auxin-responsive factor AUX/IAA-related |
| Glyma.18G270900 | 0.62 | | 0.001 | FER Malectin/receptor-like protein kinase family protein |
| Glyma.10G017600 | 0.62 | | 0.041 | AtLEA5,SAG21 senescence-associated gene 21 |
| Glyma.08G248900 | 0.63 | | 0.001 | FER Malectin/receptor-like protein kinase family protein |
| Glyma.01G128700 | 0.63 | | 0.000 | ATSCAR2,DIS3,ITB1,SCAR2,WAVE4 SCAR homolog 2 |
| Glyma.10G066000 | 0.63 | | 0.000 | AtCTR1,CTR1,SIS1 Protein kinase superfamily protein |
| Glyma.18G271200 | 0.63 | | 0.016 | FER Malectin/receptor-like protein kinase family protein |
| Glyma.11G020700 | 0.69 | | 0.015 | ATRBOH F,ATRBOHF,RBOH F,RBOHAP108,RBOHF respiratory burst oxidase protein F |
| Glyma.18G269900 | 0.70 | | 0.003 | FER Malectin/receptor-like protein kinase family protein |
| Glyma.13G362000 | 0.73 | | 0.001 | ABCG40,ATABCG40,ATPDR12,PDR12 pleiotropic drug resistance 12 |
| Glyma.18G270800 | 0.73 | | 0.004 | FER Malectin/receptor-like protein kinase family protein |
| Glyma.09G212100 | 0.73 | | 0.000 | EDR2 ENHANCED DISEASE RESISTANCE 2 |
| Glyma.02G017100 | 0.73 | | 0.014 | AtLEA5,SAG21 senescence-associated gene 21 |
| Glyma.18G271000 | 0.76 | | 0.003 | FER Malectin/receptor-like protein kinase family protein |
| Glyma.18G270100 | 0.86 | | 0.001 | FER Malectin/receptor-like protein kinase family protein |
| Glyma.05G221300 | 0.87 | | 0.000 | ARF7,BIP,IAA21,IAA23,IAA25,MSG1,NPH4,TIR5 Transcriptional factor B3 family protein / auxin-responsive factor AUX/IAA-related |
| Glyma.14G127800 | 0.97 | | 0.000 | ABCG40,ATABCG40,ATPDR12,PDR12 pleiotropic drug resistance 12 |
| Glyma.13G053700 | 1.25 | | 0.000 | FER Malectin/receptor-like protein kinase family protein |
| Glyma.03G168200 | 1.27 | | 0.009 | ABCG40,ATABCG40,ATPDR12,PDR12 pleiotropic drug resistance 12 |
| Glyma.01G222700 | 1.61 | | 0.000 | ATRBOH F,ATRBOHF,RBOH F,RBOHAP108,RBOHF respiratory burst oxidase protein F |
| Glyma.02G196000 | 1.62 | | 0.000 | FER Malectin/receptor-like protein kinase family protein |
| Glyma.18G209100 | 1.75 | | 0.000 | ATSCAR2,DIS3,ITB1,SCAR2,WAVE4 SCAR homolog 2 |
| Glyma.14G092800 | 2.31 | | 0.000 | GAMMA-VPE,GAMMAVPE gamma vacuolar processing enzyme |
| Glyma.17G230700 | 3.25 | | 0.000 | GAMMA-VPE,GAMMAVPE gamma vacuolar processing enzyme |
| **GO:0009751 response to salicylic acid** | | | | |
| **ID** | **log_2_FC** | | **padj** | **Description** |
| Glyma.08G235400 | -6.88 | | 0.000 | ATKTI1,KTI1 kunitz trypsin inhibitor 1 |
| Glyma.07G033500 | -5.22 | | 0.000 | ABCG40,ATABCG40,ATPDR12,PDR12 pleiotropic drug resistance 12 |
| Glyma.15G012000 | -5.12 | | 0.000 | ABCG40,ATABCG40,ATPDR12,PDR12 pleiotropic drug resistance 12 |
| Glyma.08G342100 | -4.20 | | 0.002 | ATKTI1,KTI1 kunitz trypsin inhibitor 1 |
| Glyma.08G341700 | -3.36 | | 0.000 | ATKTI1,KTI1 kunitz trypsin inhibitor 1 |
| Glyma.08G341800 | -3.36 | | 0.001 | ATKTI1,KTI1 kunitz trypsin inhibitor 1 |
| Glyma.14G103100 | -3.34 | | 0.000 | ATWRKY40,WRKY40 WRKY DNA-binding protein 40 |
| Glyma.05G161600 | -3.30 | | 0.000 | ATGSTU7,GST25,GSTU7 glutathione S-transferase tau 7 |
| Glyma.18G273200 | -3.28 | | 0.000 | DMR6 2-oxoglutarate (2OG) and Fe(II)-dependent oxygenase  superfamily protein |
| Glyma.18G185400 | -3.02 | | 0.001 | RLK receptor lectin kinase |
| Glyma.14G102900 | -2.70 | | 0.001 | ATWRKY40,WRKY40 WRKY DNA-binding protein 40 |
| Glyma.08G341600 | -2.62 | | 0.000 | ATKTI1,KTI1 kunitz trypsin inhibitor 1 |
| Glyma.03G168000 | -2.61 | | 0.005 | ABCG40,ATABCG40,ATPDR12,PDR12 pleiotropic drug resistance 12 |
| Glyma.08G342300 | -2.52 | | 0.031 | ATKTI1,KTI1 kunitz trypsin inhibitor 1 |
| Glyma.13G370100 | -2.50 | | 0.000 | ATWRKY40,WRKY40 WRKY DNA-binding protein 40 |
| Glyma.04G238300 | -2.41 | | 0.002 | ATWRKY30,WRKY30 WRKY DNA-binding protein 30 |
| Glyma.08G118700 | -2.31 | | 0.004 | ATGSTU7,GST25,GSTU7 glutathione S-transferase tau 7 |
| Glyma.10G005300 | -1.88 | | 0.002 | ATPTR3,PTR3 peptide transporter 3 |
| Glyma.17G126400 | -1.82 | | 0.000 | ATBT2,BT2 BTB and TAZ domain protein 2 |
| Glyma.08G118900 | -1.82 | | 0.018 | ATGSTU7,GST25,GSTU7 glutathione S-transferase tau 7 |
| Glyma.08G218600 | -1.66 | | 0.009 | ATWRKY40,WRKY40 WRKY DNA-binding protein 40 |
| Glyma.06G061900 | -1.63 | | 0.019 | ATWRKY40,WRKY40 WRKY DNA-binding protein 40 |
| Glyma.07G140000 | -1.49 | | 0.014 | ATGSTU7,GST25,GSTU7 glutathione S-transferase tau 7 |
| Glyma.13G361900 | -1.45 | | 0.000 | ABCG40,ATABCG40,ATPDR12,PDR12 pleiotropic drug resistance 12 |
| Glyma.18G294400 | -1.38 | | 0.029 | CRK5,RLK6 cysteine-rich RLK (RECEPTOR-like protein kinase) 5 |
| Glyma.11G228100 | -1.34 | | 0.042 | ATHSPRO2,HSPRO2 ortholog of sugar beet HS1 PRO-1 2 |
| Glyma.07G140200 | -0.93 | | 0.013 | ATGSTU7,GST25,GSTU7 glutathione S-transferase tau 7 |
| Glyma.08G330300 | -0.73 | | 0.001 | AHG2,ATPARN Polynucleotidyl transferase, ribonuclease H-like superfamily protein |
| Glyma.16G119000 | -0.72 | | 0.000 | SSL5,YLS2 Calcium-dependent phosphotriesterase superfamily protein |
| Glyma.17G091500 | -0.72 | | 0.002 | RDR2,SMD1 RNA-dependent RNA polymerase 2 |
| Glyma.05G168600 | -0.60 | | 0.037 | EBS1,PSL2,UGGT UDP-glucose:glycoprotein glucosyltransferases;transferases, transferring hexosyl  groups;transferases, transferring glycosyl groups |
| Glyma.08G187500 | -0.44 | | 0.001 | EBS1,PSL2,UGGT UDP-glucose:glycoprotein glucosyltransferases;transferases, transferring hexosyl  groups;transferases, transferring glycosyl groups |
| Glyma.18G269500 | 0.32 | | 0.004 | HRT,RCY1,RPP8 Disease resistance protein (CC-NBS-LRR class) family |
| Glyma.08G240800 | 0.37 | | 0.001 | WRKY4 WRKY DNA-binding protein 4 |
| Glyma.01G147800 | 0.42 | | 0.000 | EDR2 ENHANCED DISEASE RESISTANCE 2 |
| Glyma.05G106000 | 0.57 | | 0.003 | ATTBP3,ATTRB2,TBP3,TRB2 Homeodomain-like/winged-helix DNA-binding family protein |
| Glyma.06G128100 | 0.65 | | 0.000 | 2-oxoglutarate (2OG) and Fe(II)-dependent oxygenase superfamily protein |
| Glyma.03G081900 | 0.70 | | 0.013 | AS1,ATMYB91,ATPHAN,MYB91 myb-like HTH transcriptional regulator family protein |
| Glyma.13G362000 | 0.73 | | 0.001 | ABCG40,ATABCG40,ATPDR12,PDR12 pleiotropic drug resistance 12 |
| Glyma.09G212100 | 0.73 | | 0.000 | EDR2 ENHANCED DISEASE RESISTANCE 2 |
| Glyma.02G086100 | 0.87 | | 0.000 | ATRDRP1,RDR1 RNA-dependent RNA polymerase 1 |
| Glyma.14G127800 | 0.97 | | 0.000 | ABCG40,ATABCG40,ATPDR12,PDR12 pleiotropic drug resistance 12 |
| Glyma.03G261600 | 1.02 | | 0.003 | 2-oxoglutarate (2OG) and Fe(II)-dependent oxygenase superfamily protein |
| Glyma.20G206300 | 1.11 | | 0.000 | ATPTR3,PTR3 peptide transporter 3 |
| Glyma.10G183900 | 1.12 | | 0.000 | ATPTR3,PTR3 peptide transporter 3 |
| Glyma.14G223600 | 1.16 | | 0.000 | ATOPR1,OPR1 12-oxophytodienoate reductase 1 |
| Glyma.03G168200 | 1.27 | | 0.009 | ABCG40,ATABCG40,ATPDR12,PDR12 pleiotropic drug resistance 12 |
| Glyma.04G209700 | 1.43 | | 0.000 | ATPAD4,PAD4 alpha/beta-Hydrolases superfamily protein |
| Glyma.16G111800 | 1.52 | | 0.000 | ATIRE1-2,IRE1-2,IRE1A Endoribonuclease/protein kinase IRE1-like |
| Glyma.06G137000 | 1.53 | | 0.001 | DMR6 2-oxoglutarate (2OG) and Fe(II)-dependent oxygenase  superfamily protein |
| Glyma.04G190900 | 1.64 | | 0.013 | ATBT2,BT2 BTB and TAZ domain protein 2 |
| Glyma.06G156300 | 1.83 | | 0.000 | ATPAD4,PAD4 alpha/beta-Hydrolases superfamily protein |
| Glyma.14G178500 | 1.98 | | 0.000 | 2-oxoglutarate (2OG) and Fe(II)-dependent oxygenase superfamily protein |
| Glyma.14G092800 | 2.31 | | 0.000 | GAMMA-VPE,GAMMAVPE gamma vacuolar processing enzyme |
| Glyma.01G193300 | 3.24 | | 0.000 | ATBT4,BT4 BTB and TAZ domain protein 4 |
| Glyma.17G230700 | 3.25 | | 0.000 | GAMMA-VPE,GAMMAVPE gamma vacuolar processing enzyme |
| **GO:0009835 fruit ripening** | | | | |
| **ID** | **log_2_FC** | | **padj** | **Description** |
| Glyma.02G268000 | 1.36 | | 0.000 | ACO4,EAT1,EFE ethylene-forming enzyme |
| Glyma.02G268200 | -2.43 | | 0.043 | ACO4,EAT1,EFE ethylene-forming enzyme |
| Glyma.04G097900 | -3.29 | | 0.002 | MATE efflux family protein |
| Glyma.07G229100 | -1.54 | | 0.003 | ANAC029,ATNAP,NAP NAC-like, activated by AP3/PI |
| Glyma.08G018000 | -1.24 | | 0.024 | ACS6,ATACS6 1-aminocyclopropane-1-carboxylic acid (acc) synthase 6 |
| Glyma.17G009800 | -2.92 | | 0.001 | ACO4,EAT1,EFE ethylene-forming enzyme |
| Glyma.17G247400 | -2.22 | | 0.005 | MATE efflux family protein |
| **GO:0010150 leaf senescence** | | | | |
| **ID** | **log_2_FC** | | **padj** | **Description** |
| Glyma.08G092800 | -3.96 | | 0.000 | ATSRG1,SRG1 senescence-related gene 1 |
| Glyma.08G180600 | -2.29 | | 0.030 | ALD1 AGD2-like defense response protein 1 |
| Glyma.02G244000 | -2.25 | | 0.030 | ATGLN1;1,ATGSR1,GLN1;1,GSR 1 glutamine synthase clone R1 |
| Glyma.16G031900 | -2.17 | | 0.004 | AtWRKY22,WRKY22 WRKY family transcription factor |
| Glyma.01G056100 | -1.99 | | 0.001 | ATSRG1,SRG1 senescence-related gene 1 |
| Glyma.07G229100 | -1.54 | | 0.003 | ANAC029,ATNAP,NAP NAC-like, activated by AP3/PI |
| Glyma.01G068200 | -1.31 | | 0.006 | ATSRG1,SRG1 senescence-related gene 1 |
| Glyma.08G049000 | -1.26 | | 0.000 | AHK4,ATCRE1,CRE1,WOL,WOL1 CHASE domain containing histidine kinase protein |
| Glyma.02G124700 | -0.97 | | 0.049 | ATSRG1,SRG1 senescence-related gene 1 |
| Glyma.17G073800 | -0.92 | | 0.021 | Cytosol aminopeptidase family protein |
| Glyma.03G165500 | -0.62 | | 0.030 | ATPSAT1,PSAT1 phospholipid sterol acyl transferase 1 |
| Glyma.04G080700 | -0.58 | | 0.001 | ASP3,YLS4 aspartate aminotransferase 3 |
| Glyma.01G142300 | -0.39 | | 0.020 | DHHC-type zinc finger family protein |
| Glyma.18G220400 | -0.38 | | 0.018 | DHHC-type zinc finger family protein |
| Glyma.02G204000 | -0.33 | | 0.021 | Cytosol aminopeptidase family protein |
| Glyma.12G159600 | -0.27 | | 0.045 | RNA-binding (RRM/RBD/RNP motifs) family protein |
| Glyma.14G040800 | -0.25 | | 0.027 | RNA-binding (RRM/RBD/RNP motifs) family protein |
| Glyma.13G060400 | 0.20 | | 0.037 | AKIN10,KIN10,SNRK1.1 SNF1 kinase homolog 10 |
| Glyma.12G164100 | 0.21 | | 0.019 | ARF1 auxin response factor 1 |
| Glyma.07G211600 | 0.28 | | 0.035 | ATATG2,ATG2 autophagy 2 |
| Glyma.13G145100 | 0.28 | | 0.004 | ATEIN2,CKR1,EIN2,ERA3,ORE2,ORE3,PIR2 NRAMP metal ion transporter family protein |
| Glyma.17G008400 | 0.30 | | 0.037 | UPL5 ubiquitin protein ligase 5 |
| Glyma.02G133400 | 0.32 | | 0.024 | ATATG2,ATG2 autophagy 2 |
| Glyma.11G012000 | 0.39 | | 0.009 | AT-RSH3,ATRSH3,RSH3 RELA/SPOT homolog 3 |
| Glyma.07G265600 | 0.39 | | 0.037 | UPL5 ubiquitin protein ligase 5 |
| Glyma.16G015700 | 0.40 | | 0.038 | ORMDL family protein |
| Glyma.03G118800 | 0.42 | | 0.019 | RNA-binding (RRM/RBD/RNP motifs) family protein |
| Glyma.16G008900 | 0.45 | | 0.000 | Subtilase family protein |
| Glyma.02G085900 | 0.49 | | 0.006 | ARR2,RR2 response regulator 2 |
| Glyma.02G046900 | 0.49 | | 0.000 | ATMCB1,MBP1,MCB1,RPN10 regulatory particle non-ATPase 10 |
| Glyma.03G216400 | 0.52 | | 0.027 | AT-RSH1,ATRSH1,RSH1 RELA/SPOT homolog 1 |
| Glyma.07G205500 | 0.58 | | 0.000 | RNA-binding (RRM/RBD/RNP motifs) family protein |
| Glyma.02G249600 | 0.60 | | 0.005 | RCA rubisco activase |
| Glyma.07G243300 | 0.60 | | 0.005 | ARR2,RR2 response regulator 2 |
| Glyma.10G017600 | 0.62 | | 0.041 | AtLEA5,SAG21 senescence-associated gene 21 |
| Glyma.19G212900 | 0.63 | | 0.007 | AT-RSH1,ATRSH1,RSH1 RELA/SPOT homolog 1 |
| Glyma.07G040100 | 0.63 | | 0.000 | Subtilase family protein |
| Glyma.06G128100 | 0.65 | | 0.000 | 2-oxoglutarate (2OG) and Fe(II)-dependent oxygenase superfamily protein |
| Glyma.05G137500 | 0.66 | | 0.026 | ATSRG1,SRG1 senescence-related gene 1 |
| Glyma.02G017100 | 0.73 | | 0.014 | AtLEA5,SAG21 senescence-associated gene 21 |
| Glyma.11G215500 | 0.78 | | 0.020 | ATGLN1;1,ATGSR1,GLN1;1,GSR 1 glutamine synthase clone R1 |
| Glyma.07G051600 | 0.82 | | 0.000 | ATCRK3,CRK3 CDPK-related kinase 3 |
| Glyma.14G067000 | 0.86 | | 0.000 | RCA rubisco activase |
| Glyma.16G008400 | 0.94 | | 0.000 | AT-RSH3,ATRSH3,RSH3 RELA/SPOT homolog 3 |
| Glyma.03G261600 | 1.02 | | 0.003 | 2-oxoglutarate (2OG) and Fe(II)-dependent oxygenase superfamily protein |
| Glyma.18G260000 | 1.08 | | 0.024 | NRT1.5 nitrate transporter 1.5 |
| Glyma.11G221000 | 1.09 | | 0.001 | RCA rubisco activase |
| Glyma.04G140100 | 1.11 | | 0.000 | ATPUB44,PUB44,SAUL1 senescence-associated E3 ubiquitin ligase 1 |
| Glyma.14G223600 | 1.16 | | 0.000 | ATOPR1,OPR1 12-oxophytodienoate reductase 1 |
| Glyma.07G039600 | 1.18 | | 0.000 | AT-RSH3,ATRSH3,RSH3 RELA/SPOT homolog 3 |
| Glyma.05G070600 | 1.19 | | 0.000 | NRT1.5 nitrate transporter 1.5 |
| Glyma.16G051800 | 1.19 | | 0.004 | ANAC083,NAC083,VNI2 NAC domain containing protein 83 |
| Glyma.18G036400 | 1.20 | | 0.001 | RCA rubisco activase |
| Glyma.04G209700 | 1.43 | | 0.000 | ATPAD4,PAD4 alpha/beta-Hydrolases superfamily protein |
| Glyma.17G153300 | 1.47 | | 0.000 | NRT1.5 nitrate transporter 1.5 |
| Glyma.15G240600 | 1.50 | | 0.000 | ATSRG1,SRG1 senescence-related gene 1 |
| Glyma.06G156300 | 1.83 | | 0.000 | ATPAD4,PAD4 alpha/beta-Hydrolases superfamily protein |
| Glyma.12G040400 | 1.90 | | 0.000 | ARM repeat superfamily protein |
| Glyma.14G178500 | 1.98 | | 0.000 | 2-oxoglutarate (2OG) and Fe(II)-dependent oxygenase superfamily  protein |
| Glyma.08G240400 | 2.30 | | 0.000 | AKIN10,KIN10,SNRK1.1 SNF1 kinase homolog 10 |
| Glyma.14G092800 | 2.31 | | 0.000 | GAMMA-VPE,GAMMAVPE gamma vacuolar processing enzyme |
| Glyma.17G230700 | 3.25 | | 0.000 | GAMMA-VPE,GAMMAVPE gamma vacuolar processing enzyme |
| Glyma.09G237600 | 3.32 | | 0.000 | NRT1.5 nitrate transporter 1.5 |
| **GO:0012501 programmed cell death** | | | | |
| **ID** | **log_2_FC** | | **padj** | **Description** |
| Glyma.08G235400 | -6.88 | | 0.000 | ATKTI1,KTI1 kunitz trypsin inhibitor 1 |
| Glyma.08G342100 | -4.20 | | 0.002 | ATKTI1,KTI1 kunitz trypsin inhibitor 1 |
| Glyma.16G056300 | -4.02 | | 0.000 | ATTSO2,TSO2 Ferritin/ribonucleotide reductase-like family protein |
| Glyma.08G341700 | -3.36 | | 0.000 | ATKTI1,KTI1 kunitz trypsin inhibitor 1 |
| Glyma.08G341800 | -3.36 | | 0.001 | ATKTI1,KTI1 kunitz trypsin inhibitor 1 |
| Glyma.08G341600 | -2.62 | | 0.000 | ATKTI1,KTI1 kunitz trypsin inhibitor 1 |
| Glyma.08G342300 | -2.52 | | 0.031 | ATKTI1,KTI1 kunitz trypsin inhibitor 1 |
| Glyma.03G195600 | -1.71 | | 0.031 | ATTSO2,TSO2 Ferritin/ribonucleotide reductase-like family protein |
| Glyma.18G294400 | -1.38 | | 0.029 | CRK5,RLK6 cysteine-rich RLK (RECEPTOR-like protein kinase) 5 |
| Glyma.08G033300 | -0.68 | | 0.000 | ATHXK1,GIN2,HXK1 hexokinase 1 |
| Glyma.05G226600 | -0.42 | | 0.042 | ATHXK1,GIN2,HXK1 hexokinase 1 |
| Glyma.19G242400 | 0.45 | | 0.002 | AIN1,ATXRN4,EIN5,XRN4 exoribonuclease 4 |
| Glyma.14G218000 | 0.58 | | 0.000 | AtXRN3,XRN3 5\`-3\` exoribonuclease 3 |
| **GO:1900055 regulation of leaf senescence** | | | | |
| **ID** | **log_2_FC** | | **padj** | **Description** |
| Glyma.01G142300 | -0.39 | | 0.020 | DHHC-type zinc finger family protein |
| Glyma.18G220400 | -0.38 | | 0.018 | DHHC-type zinc finger family protein |
| **GO:1900056 negative regulation of leaf senescence** | | | | |
| **ID** | **log_2_FC** | | **padj** | **Description** |
| Glyma.09G065900 | -1.44 | | 0.000 | MMP matrix metalloproteinase |
| Glyma.01G147800 | 0.42 | | 0.000 | EDR2 ENHANCED DISEASE RESISTANCE 2 |
| Glyma.09G212100 | 0.73 | | 0.000 | EDR2 ENHANCED DISEASE RESISTANCE 2 |
| Glyma.08G294300 | 0.75 | | 0.009 | Galactosyltransferase family protein |
| Glyma.08G292300 | 1.24 | | 0.001 | MMP matrix metalloproteinase |
| Glyma.18G128600 | 1.42 | | 0.000 | Galactosyltransferase family protein |

Table S3 - Examples of genes differentially expressed shared by [35S:GmNAC81 (20DAG) - BR16 (20DAG)] vs [BR16 (80 DAG) - BR16 (20DAG)].

| ID | 35S:GmNAC81 (20DAG) - BR16 (20DAG) | | BR16 (80 DAG) –  BR16 (20DAG) | | Description |
| --- | --- | --- | --- | --- | --- |
|  | log_2_FC | padj | log_2_FC | padj |  |
| Glyma.08G240400 | 2.30 | 0.000 | 3.51 | 0.000 | SNF1 kinase homolog 10 |
| Glyma.09G237600 | 3.32 | 0.000 | 4.10 | 0.000 | Nitrate transporter 1.5 |
| Glyma.14G072300 | 3.48 | 0.000 | 2.89 | 0.000 | Jasmonate O-methyltransferase |
| Glyma.19G179900 | 4.36 | 0.000 | 6.57 | 0.000 | Seven transmembrane MLO family protein |
| Glyma.08G235400 | -6.88 | 0.000 | -3.76 | 0.000 | Kunitz trypsin inhibitor 1 |
| Glyma.10G184700 | -5.17 | 0.000 | -5.11 | 0.000 | Serine protease inhibitor, |

Table S4 - Candidate targets of GmNAC081 harboring the binding site TGTG[T/G/C] in 5' flanking sequences.

| Gene | Locus | Frequency of TGTGT(TGC) | Distance from ATG | Probability |
| --- | --- | --- | --- | --- |
| MLO3 | Glyma.19G179900 | 2 | -367; -1977 | 0.1824 |
| JMT | Glyma.14G072300 | 1 | -316 | 0.4300 |
| NRT1.5 | Glyma.09G237600 | 5 | -1032; -1034; -1036; -1538; -1635 | 0.0039 |
| KIN10 | Glyma.08G240400 | 3 | - 793; -1254; -1906 | 0.0613 |
| KTI1 | Glyma.08G235400 | 2 | - 1179; -1817 | 0.1824 |
| SPI | Glyma.10G184700 | 1 | -1535 | 0.4300 |

Table S5 - Genes differentially expressed involved in drought stress between 35S:GmNAC81 and BR16 plants at 20 DAG.

| **ID** | **log_2_FC** | **padj** | **Description** |
| --- | --- | --- | --- |
| Glyma.11G038800 | -1.28 | 0.000 | ATOST1,OST1,P44,SNRK2-6,SNRK2.6,SRK2E Protein kinase  superfamily protein |
| Glyma.05G021100 | -1.17 | 0.042 | ATRBOH F,ATRBOHF,RBOH F,RBOHAP108,RBOHF respiratory burst oxidase protein F |
| Glyma.08G018900 | -1.05 | 0.025 | ATRBOH F,ATRBOHF,RBOH F,RBOHAP108,RBOHF respiratory burst oxidase protein F |
| Glyma.15G250100 | 1.27 | 0.045 | ATNCED3,NCED3,SIS7,STO1 nine-cis-epoxycarotenoid dioxygenase 3 |
| Glyma.08G176300 | 1.49 | 0.016 | ATNCED3,NCED3,SIS7,STO1 nine-cis-epoxycarotenoid dioxygenase 3 |
| Glyma.01G222700 | 1.61 | 0.000 | ATRBOH F,ATRBOHF,RBOH F,RBOHAP108,RBOHF respiratory burst oxidase protein F |
| Glyma.01G153300 | -1.42 | 0.045 | CYP707A1 cytochrome P450, family 707, subfamily A, polypeptide 1 |
| Glyma.01G204200 | -1.46 | 0.010 | ATOST1,OST1,P44,SNRK2-6,SNRK2.6,SRK2E Protein kinase  superfamily protein |
| Glyma.02G017200 | -1.12 | 0.002 | APP,ATPARP1,PARP1,PP poly(ADP-ribose) polymerase |
| Glyma.02G046600 | 1.12 | 0.000 | AHK1,ATHK1,HK1 histidine kinase 1 |
| Glyma.02G128400 | -1.01 | 0.046 | DOR F-box and associated interaction domains-containing protein |
| Glyma.03G006600 | 1.14 | 0.000 | AtMYB60,MYB60 myb domain protein 60 |
| Glyma.03G161300 | -1.71 | 0.000 | ATPARP2,PARP2 poly(ADP-ribose) polymerase 2 |
| Glyma.03G168000 | -2.61 | 0.005 | ABCG40,ATABCG40,ATPDR12,PDR12 pleiotropic drug resistance 12 |
| Glyma.03G168200 | 1.27 | 0.009 | ABCG40,ATABCG40,ATPDR12,PDR12 pleiotropic drug resistance 12 |
| Glyma.03G180900 | -1.55 | 0.001 | PIP2,PIP2;1,PIP2A plasma membrane intrinsic protein 2A |
| Glyma.03G229800 | -2.89 | 0.000 | AtGolS2,GolS2 galactinol synthase 2 |
| Glyma.04G044900 | -1.97 | 0.004 | STZ,ZAT10 salt tolerance zinc finger |
| Glyma.04G048500 | 2.10 | 0.000 | SQE1,XF1 FAD/NAD(P)-binding oxidoreductase family protein |
| Glyma.04G056600 | -1.13 | 0.015 | ATGPA1,GP ALPHA 1,GPA1 G protein alpha subunit 1 |
| Glyma.05G208700 | -2.76 | 0.000 | PIP1;4,PIP1E,TMP-C plasma membrane intrinsic protein 1;4 |
| Glyma.05G225900 | -2.60 | 0.000 | PTHR33172,PTHR33172:SF2 AT5G56550.1 ATOXS3,OXS3 oxidative stress 3 |
| Glyma.06G045400 | -2.32 | 0.000 | STZ,ZAT10 salt tolerance zinc finger |
| Glyma.07G033500 | -5.22 | 0.000 | ABCG40,ATABCG40,ATPDR12,PDR12 pleiotropic drug resistance 12 |
| Glyma.08G032800 | -1.53 | 0.039 | PTHR33172,PTHR33172:SF2 AT5G56550.1 ATOXS3,OXS3 oxidative stress 3 |
| Glyma.08G120800 | 1.12 | 0.048 | ATTPS1,TPS1 trehalose-6-phosphate synthase |
| Glyma.09G147200 | -3.47 | 0.004 | CBF4,DREB1D C-repeat-binding factor 4 |
| Glyma.09G157900 | -1.38 | 0.000 | ATCLC-C,CLC-C chloride channel C |
| Glyma.09G203500 | 1.54 | 0.000 | PCK1,PEPCK phosphoenolpyruvate carboxykinase 1 |
| Glyma.11G149900 | -1.70 | 0.000 | PTHR33790,PTHR33790:SF1 AT2G41430.1 CID1,ERD15,LSR1 dehydration-induced protein (ERD15) |
| Glyma.11G152700 | -2.75 | 0.000 | PF13967,PF02714 PTHR13018,PTHR13018:SF30 GO:GO:0016020 AT3G54510.2 Early-responsive to dehydration stress protein (ERD4) |
| Glyma.11G208700 | -2.70 | 0.000 | PTHR33172,PTHR33172:SF8 AT5G56550.1 ATOXS3,OXS3 oxidative stress 3 |
| Glyma.11G230100 | -2.46 | 0.000 | ATPLC2,PLC2 phospholipase C 2 |
| Glyma.12G084700 | -1.42 | 0.000 | HD2C,HDT3 histone deacetylase 2C |
| Glyma.12G132100 | 1.36 | 0.014 | ATSYP61,OSM1,SYP61 syntaxin of plants 61 |
| Glyma.12G181400 | -1.11 | 0.000 | HD2C,HDT3 histone deacetylase 2C |
| Glyma.13G088700 | -2.92 | 0.000 | ANNAT1,ATOXY5,OXY5 annexin 1 |
| Glyma.13G319500 | -1.28 | 0.000 | HD2C,HDT3 histone deacetylase 2C |
| Glyma.13G361900 | -1.45 | 0.000 | ABCG40,ATABCG40,ATPDR12,PDR12 pleiotropic drug resistance 12 |
| Glyma.14G090400 | 2.59 | 0.000 | SQE1,XF1 FAD/NAD(P)-binding oxidoreductase family protein |
| Glyma.14G098000 | -1.25 | 0.003 | ATGPA1,GP ALPHA 1,GPA1 G protein alpha subunit 1 |
| Glyma.14G218400 | -1.28 | 0.000 | PF03134 PTHR12300,PTHR12300:SF26 AT5G42560.1 Abscisic acid-responsive (TB2/DP1, HVA22) family protein |
| Glyma.15G012000 | -5.12 | 0.000 | ABCG40,ATABCG40,ATPDR12,PDR12 pleiotropic drug resistance 12 |
| Glyma.15G238400 | -1.22 | 0.023 | ANNAT4 annexin 4 |
| Glyma.17G013800 | -1.30 | 0.000 | CPK21 calcium-dependent protein kinase 21 |
| Glyma.17G050600 | 1.05 | 0.000 | PF07876 PTHR33178,PTHR33178:SF3 AT2G31670.1 Stress responsive alpha-beta barrel domain protein |
| Glyma.17G053100 | -1.16 | 0.000 | PF00400 PTHR22847,PTHR22847:SF425 KOG0649 GO:GO:0005515 AT2G19430.1 AtTHO6,DWA1,THO6 DWD (DDB1-binding WD40 protein) hypersensitive to ABA 1 |
| Glyma.18G027100 | -1.44 | 0.001 | ATPLC2,PLC2 phospholipase C 2 |
| Glyma.18G045200 | -1.84 | 0.002 | PTHR33172,PTHR33172:SF8 AT5G56550.1 ATOXS3,OXS3 oxidative stress 3 |
| Glyma.18G118100 | -1.05 | 0.000 | SQE1,XF1 FAD/NAD(P)-binding oxidoreductase family protein |
| Glyma.19G227800 | -3.33 | 0.000 | AtGolS2,GolS2 galactinol synthase 2 |
| Glyma.20G066800 | -1.48 | 0.000 | PF14703,PF13967,PF02714 PTHR13018,PTHR13018:SF47 GO:GO:0016020 AT4G22120.6 ERD (early-responsive to dehydration stress) family protein |
| Glyma.20G102000 | -2.14 | 0.002 | PF02893 PTHR31969,PTHR31969:SF1 AT5G23370.1 GRAM domain-containing protein / ABA-responsive protein-related |
| Glyma.20G102100 | -1.53 | 0.046 | PF02893 PTHR31969,PTHR31969:SF1 AT5G08350.1 GRAM domain-containing protein / ABA-responsive protein-related |

Table S6 - List of oligonucleotides used in the qRT-PCR assays.

| Primer | Sequence (5’—3’) | Gene |
| --- | --- | --- |
| GM UKN2 F | GCCTCTGGATACCTGCTCAAG | Unknown 2 |
| GM UKN2 R | ACCTCCTCCTCAAACTCCTCTG |  |
| HELIC Fwd | TAACCCTAG CCCCTTCGC CT | HELICASE |
| HELIC Rvs | GCCTTGTCGTCTTCCTCCTCG |  |
| qRTGmNAC081 | CCA ACA AAA GCA CTT GTG GCA | GmNAC081 |
| qRTGmNAC081 | GGA CTA TTC AACTGA GCCCAAAAG |  |
| qRTGmMLO3Fwd | AGTGGCACAGATGGGTTCAG | MLO3 |
| qRTGmMLO3Rv | TCTTGGCTCCATTCTGAGGAC |  |
| qRTGmNRT1.5Fwd | AATGCACAAACACCTGATGG | NRT1.5 |
| qRTGmNRT1.5Rv | ATCCATCCTGGCATCTCATC |  |
| qRTGmJMTFwd | GGAAGGGTCATTCATTGTGG | JMT |
| qRTGmJMTRv | ATGGTCCTTGCAACTCGTTC |  |
| qRTGmKIN10Fwd | TTGATGTCTGGAGTTGTGGTG | KIN10 |
| qRTGmKIN10Rv | TGGCTCCAGGAGATAGATGG |  |
| qRTGmKIT1Fwd | TTGCCCTCAACGTTCAAGATG | KIT1 |
| qRTGmKIT1RV | TTCCACTCCTCGTGTCGTTC |  |
| qRTGmSPIFwd | GGCCTGAGCTAGTTGGAGTG | SPI |
| qRTGmSPIRv | CAATGTGGGGAACTTCCTTG |  |
