# Supplementary material for: GmNAC81 Inversely Modulates Leaf Senescence and Drought Tolerance
Source: Front Genet. 2020 Nov 24;11:601876. doi: 10.3389/fgene.2020.601876 (PMC7732657; doi:10.3389/fgene.2020.601876)
Supplement: Supplementary Methods — Detailed description of Materials and Methods. [file Data_Sheet_3.docx]

**Supplementary Methods**

**Plant growth and PCR-based diagnostic of transgenic lines**

Soybean plants (*Glycine max* cv. BR16) transformed with the gene GmNAC81, strains NAC081-1, and NAC081-3, have been previously described (Pimenta et al., 2016). The transgenics lines were confirmed by PCR for gene insertion and RT-qPCR for transgene expression. The genomic DNA was isolated from 7-day leaves of soybean using CTAB extraction buffer 2% (w/v), 1.4 M NaCl, 100 mM Tris-HCl pH 8.0, 20 mM EDTA and ß-mercaptoethanol 0.2% (v/v), as described (Doyle and Doyle, 1987). PCR was performed using 50 ng of genomic DNA as a template, 0.5 μM of each of the 35AMVF – CCACTATCCTTCGCAAGAC and NAC6R – TCTCTCTCTTCCTCTAGTGCTCG primers and 0.25 U of GoTaq® DNA Polymerase Promega, in a final volume of 50 μL. The mixture was heated to 94 °C for 3 minutes, and PCR was conducted for 35 cycles (45s to 94 °C, 45s to 53 °C and 60s to 72 °C) with a final extension at 72 °C, for 10 min, using a C1000™ Thermal Cycler system (BIO-RAD). The PCR product was examined by electrophoresis on agarose gel 1% (w/v) stained with ethidium bromide 1μg/ml. Transgenic seedlings were transferred to 3-liter pots containing a mixture of soil, substrate, and sand (3: 1: 1) and grown in a greenhouse under natural conditions of light, relative humidity (65-85%) and temperature (15-35 °C).

**RNA extraction and cDNA synthesis**

Total RNA was isolated from leaves by the Trizol method, according to the manufacturer’s recommendations. The integrity of the RNA was verified by electrophoresis on a denaturing agarose gel 1.2% (w/v) stained with ethidium bromide 1μg/ml. The total RNA was treated with 1U of Rnase-free DNase. For cDNA synthesis, 3 μg of RNA, the enzyme M-MLV (Invitrogen) and oligo-dT reverse transcriptase were used, according to the manufacturer’s recommendations.

**Real-time RT-PCR assays (RT-qPCR)**

The real-time PCR procedures, including efficiency tests, validations, and experiments, were conducted following the information contained in the manuals provided by Applied Biosystems. The reactions were conducted on the ABI7500 Real-Time PCR Systems (Applied Biosystems) using SYBR® Green PCR Master Mix (Applied Biosystems). Gene expression was quantified using the comparative method of Ct:2^–∆Ct^ and 2^–∆∆Ct^. For the expression of gene GmNAC81, the gene Helicase was used as an endogenous control for the normalization of data. The Unknown2 gene was used as an endogenous control for data normalization for the differentially expressed genes (Hu et al., 2009; Hirschburger et al., 2015). The oligonucleotides used for RT-qPCR are listed in Supplementary Table S6.

**RNA-Sequencing experiment**

The plant material was obtained from the third leaf (counting up from the cotyledonary leaf) of three soybean plants for each of the treatments. The treatments were the two independent strains expressing GmNAC81 and, as a control, the cultivar BR16 (WT). Leaves were collected in two developmental stages of the plant, vegetative stage three (V3; 20 days after germination, DAG) and reproductive stage six (R6; 80 DAG). Each developmental stage comprised a set of nine libraries. Each set of nine libraries was placed in two lanes, thus constituting a replica of each set. In each plant, six discs of 0.9 cm in diameter were collected in liquid nitrogen and immediately stored at −80 ° C. The total RNA was extracted using the Trizol reagent (Invitrogen), according to the manufacturer’s recommendations, followed by precipitation with isopropanol. The total RNA was then treated with the DNAse enzyme to avoid DNA contamination. The integrity, quality, and quantity of RNA extracted were monitored using the Bioanalyzer 2100 (Agilent). The sequencing libraries' preparation was performed using the TruSeq® Stranded Sample Preparation and Illumina’s Low Sample (LS) Protocol. The samples were sequenced on 100bp paired-end reads. The sequencing of paired ends libraries was performed from cDNA, using the Illumina HiSeq2500 high mode and nine libraries/lane., which were placed in duplicates on different lanes.

**Analysis of differential gene expression using RNA-seq data**

The quality of sequences (reads) was determined using FASTQC (Andrews, 2010; <http://www.bioinformatics.babraham.ac.uk/projects/fastqc/>). Then, the Sickle program (Joshi and Fass, 2011) was applied to remove low-quality sequences (Q <30). The resulting sequences were mapped using Bowtie 2 program (Langmead and Salzberg, 2012) in the soybean genome version Wm82.a2.v1 (Goodstein et al., 2012), Phytozome 12 database. The fragment count mapped in gene regions, carried out by the Rsubread package (Liao et al., 2013) present in Bioconductor/R, generated the count matrix per gene noted in the soybean genome. Differential expression was examined using DESeq2 package (Love et al., 2014). Log_2_.Fold Change > 1.0 or < - 1 and q-value ≤ 0.01 were used as the cutoff point. The quality test of the replicates related to the lanes and treatments was performed using the principal component analysis (PCA) and hierarchical grouping by the Ward method. Differentially expressed (DE) genes were stored using SQL tables in the PostgreSQL relational database ([http](https://www)://inctipp.bioagro.ufv.br/GmNac81_genome/), which listed corresponding log2FC (fold change) and p-values corrected by FDR (q-value) for all DE genes. The analyzes of gene ontology and enrichment of metabolic and signaling pathways were performed respectively by the packages Gostats (Falcon and Gentleman, 2007) and Pathview (Luo et al., 2013).

**Identification of candidate genes for direct targets of GmNAC81**

Differentially expressed genes were first confirmed by RT-qPCR. Then, 2000 bp -5' flanking sequence of each selected gene was retrieved from Phytozone. The GmNAC81 binding element, TGTG [T/G/C], was then identified and mapped. The probability of a random occurrence of this sequence was calculated using the Cumulative Binomial Distribution test for cis-elements (Haberer et al., 2006).

**Detection of H_2_O_2_ by DAB (Diaminobenzidine)**

The production of hydrogen peroxide (H_2_O_2_) was evaluated using the 3,3-diaminobenzidine reagent (DAB-Sigma), according to Weigel & Glazebrook (2002). The third leaf was collected from 80 days after germination and immediately submerged into 10 mM potassium phosphate solution (K_2_HPO_4_) containing 1 mg.mL^-1^ of DAB, pH 3.8, and incubated for 8 hours under constant light. After this period, the leaves were boiled in 100% ethanol until totally distained.

**The productivity of GmNAC81-overexpressing lines**

Four replicates of NAC081-1, NAC081-3, BR16 were grown under greenhouse conditions in two growing seasons (2015-2016 and 2016-2017). Each experimental plot consisted of four plants of each variety. The number of pods per plant, seeds per pod, seeds per plant, and the weight of seeds per plant were measured. The number of seeds and the weight of seeds per plant were used to obtain the weight of 1000 seeds. The data were submitted to no paired T-test using the software R (R Core Team, 2020).

**Induction of water deficit stress**

Plants were grown under greenhouse conditions and normal water supply until reaching the V3 stage (fully expanded third trifoliate) when the field capacity was measured in all pots. After the excess water was drained by gravity, each pot was weighed and recorded. The pots were distributed in completely randomized blocks with six treatments, irrigated and unirrigated GmNAC81-1, irrigated and unirrigated GmNAC81-3, irrigated and unirrigated control BR16. Each treatment consisted of 48 plants. All pots were weighed every day to determine the water loss by evapotranspiration, which was refilled in the irrigated treatments, considering the field capacity. In the unirrigated treatments (NI), the water was refilled every two days by a decreasing series of value lost by evapotranspiration (40; 35; 30; 25; 20; 15; 10; 5; 2.5 and 0%). The leaf water potential (ψ) was measured on the third emergent trifoliate with a Scholander Pump between 3:00 am and 5:00 am, during the stress period, until the value of ψ = –2.0. The mean values of GmNAC81 samples were compared to BR16 samples using no paired T-test in the R (R Core Team, 2020).

**Determination of the relative water content**

For this assay, 6 x 0.9 cm-leaf discs were collected in the third trifoliolate leaf (counting up from the cotyledon leaf) from four plants of each treatment. The discs were collected at 5:00 am; after determining the samples' fresh weight (FW), they were completely hydrated in Petri dishes, containing 40 ml of deionized water for 4 hours and the turgid weight (TW) was determined. After dehydration at 70 °C for 24 hours, the dry weight (DW) was determined. Relative water content (RWC) was determined using the equation RWC = (FW-DW) / (TW-DW) (Weatherley 1950). The mean values of GmNAC81 samples were compared to BR16 samples using no paired T-test in the R (R Core Team, 2020).

**Physiological parameters by gas exchange**

The Photosynthetic rate (*A*), the stomatal conductance (g_s_), the transpiration rate (E) and the intercellular CO_2_ / ambient CO_2_ (C_i_/C_a_) were evaluated in the third fully expanded leaf using an infrared gas analyzer (IRGA - portable model LI-6400xt, LI-COR Biosciences Inc., Lincon, Nebraska, USA). The measurements were carried out between 08:00 am and 11:00 am, in a greenhouse, using constant photosynthetically active radiation (PAR) (1000 μmol photons m^−2^s^−1^), CO_2_ concentration of 400 μmol CO_2_ mol^-1^, temperature and humidity ambient. The instant water use efficiency (EUW) was calculated by the A/E ratio, the carboxylation efficiency (EFC) by the A/C_i_ ratio, and the intrinsic water use efficiency (EIW) by the A/g_s_ ratio. The mean values of GmNAC81 samples were compared to BR16 samples using no paired T-test in the R (R Core Team, 2020).

**Physiological parameters by fluorescence**

The chlorophyll fluorescence variables were obtained in the same area of ​​the leaf where the gas exchange measurements were performed, with a fluorometer coupled to the IRGA (LI-6400xt, LI-COR). The parameters adapted to the dark were evaluated in the previous night (10:00 pm - 12:00 am), to ensure that the reaction centers were completely open (all primary oxidized receptors), with minimal heat loss.

The fluorescence variables obtained were the minimal fluorescence yield in dark-adapted state (F_o_), maximum fluorescence yield in dark-adapted state (F_m_) and maximum variable fluorescence yield in dark-adapted state (F_v_) was determined by the difference between F_o_ and F_m_. The potential quantum yield of PSII (F_v_ /F_m_) was calculated with the values ​​of F_v_ and F_m_ (Genty et al., 1989). The variables of the slow phase of the chlorophyll fluorescence induction were obtained sequentially after the analysis of gas exchanges with the application of an actinic illumination and a pulse of saturating actinic light to determine the following variables: maximum fluorescence yield measured when the actinic radiation is switched on (F) and maximum fluorescence yield in the light-adapted state (F_m_'). From these parameters, it was possible to calculate the minimal fluorescence yield in light-adapted state (F_o_'= F_o_ / [((F_m_ - F_o_ / F_m_) + (F_o_ / F_m_'))] (Oxborough and Baker, 1997). The non-photochemical quenching (NPQ) and effective quantum yield of photochemical energy conversion in photosystem II (Y_II_) were calculated according to Genty et al. (1989) and Hendrikson et al. (2004). The Y_II_ was used to estimate the apparent rate of electron transport at the PSII level (ETR) (Bilger et al., 1995). The mean values of GmNAC81 samples were compared to BR16 samples using no paired T-test in the R (R Core Team, 2020).

**Determination of MDA (malondialdehyde)**

Leaf lipid peroxidation was estimated through the content of TBA-reactive compounds, such as malondialdehyde (MDA), and was conducted according to Cakmak and Horst (1991). The leaf discs were collected at 5:00 am and weighed on a precision scale. The variation due to the plants' degree of hydration was corrected using the relative water content (correction factor), correcting all plants to 100% hydration. The leaves were ground in liquid nitrogen and homogenized with 2 ml of trichloroacetic acid 0.1% (v/v) and then centrifuged at 12000 g for 15 min. All steps were performed at 4 ° C. The supernatant aliquots (0.5 ml) were added to 1.5 ml of thiobarbituric acid 0.5% (v/v) and trichloroacetic acid 20% (v/v), and then incubated at 90 °C for 20 min. The reaction was stopped for incubation on ice, followed by centrifugation at 13000 g for 4 min. The absorbance of the supernatant was determined at 532 nm and subtracted from the non-specific A_600_. The concentration of malondialdehyde was calculated using the molar absorptivity coefficient of 155 mM^-1^.cm^-1^ (Heath and Packer, 1968). The mean values of GmNAC81 samples were compared to BR16 samples using no paired T-test in the R (R Core Team, 2020).

**Determination of chlorophyll and carotenoid contents**

The quantification of photosynthetic pigments was performed according to Wellburn (1994). The leaf discs were collected at 5:00 am and weighed on a precision scale. The variation due to the plants' degree of hydration was corrected using the relative water content (correction factor), correcting all plants to 100% hydration. The leaf discs were incubated with 3 ml of dimethyl sulfoxide (DMSO) saturated CaCO_3_. Chlorophylls a and b absorb spectral bands (maximum) in the blue (around 428 and 453 nm) and red (around 661 and 642 nm). The carotenoids isolated has a wide range of absorption, three peaks in the blue spectral between 400 and 500 nm. After 16 h at room temperature, the absorbance of the extracts was determined spectrophotometrically at 480, 649.1 and 665.1 nm and expressed as μg g^-1^FW. The mean values of GmNAC81 samples were compared to BR16 samples using no paired T-test in the R (R Core Team, 2020).

**References**

Andrews, S. (2010). FastQC: A Quality Control Tool for High Throughput Sequence Data. Available online at: <http://www.bioinformatics.babraham.ac.uk/projects/fastqc/>

Bilger, W., Schreiber, U., Bock, M. (1995). Determination of the quantum efficiency of photosystem II and non- photochemical quenching of chlorophyll fluorescence in the field. *Oecologia* 102: 425-432. doi: 10.1007/BF00341354

Cakmak, I., and Horst, J.H. (1991). Effects of aluminium on lipid peroxidation, superoxid dismutase, catalase and peroxidade activities in root tips of soybean (*Glycine max*). *Physiol. Plant.* 83: 463–468. doi: 10.1111/j.1399-3054.1991.tb00121.x

Doyle, J.J. and Doyle, J.L. (1987). A rapid DNA isolation procedure for small qualntities of freshleaf tissue. *Phytochemical Bulletin.* 19: 11-15.

Falcon, S., and Gentleman, R. (2007). Using GOstats to test gene lists for GO term association. *Bioinformatics* 23: 257-258. doi: 10.1093/bioinformatics/btl567

Genty, B., Briantais, J-M., Baker, N.R. (1989). The relationship between the quantum yield of photosynthetic electron transport and quenching of chlorophyll fluorescence. *Biochim. Biophys. Acta (BBA) - General Subjects* 990: 87–92. doi: 10.1016/S0304-4165(89)80016-9

Goodstein, D.M., Shu, S., Howson, R., Neupane, R., Hayes, R.D., Fazo, J., Mitros, T., Dirks, W., Hellsten, U., Putnam, N., Rokhsar, D.S. (2012). Phytozome: a comparative platform for green plant genomics. *Nucleic Acids Res*. 40: D1178-D1186. doi: 10.1093/nar/gkr944

Haberer, G., Mader, M..T, Kosarev, P., Spannagl, M., Yang, L., Mayer, K,F. (2006) Large-scale cis-element detection by analysis of correlated expression and sequence conservation between Arabidopsis and Brassica oleracea. Plant Physiol. 142:1589-1602. doi: 10.1104/pp.106.085639.

Heath, R.L., and Packer, L. (1968). Photoperoxidation in isolated chloroplasts: I. Kinetics and stoichiometry of fatty acid peroxidation. *Arch. Biochem. Biophys.* 125: 189-198. doi: 10.1016/0003-9861(68)90654-1

Hendrickson, L., Furbank, R.T., Chow, W.S. (2004). A simple alternative approach to assessing the fate of absorbed light energy using chlorophyll fluorescence. *Photosynth. Res.* 82: 73–81. doi: 10.1023/B:PRES.0000040446.87305.f4

Hirschburger, D., Müller, M., Voegele, R.T., Link, T. (2015). Reference Genes in the Pathosystem Phakopsora pachyrhizi/ Soybean Suitable for Normalization in Transcript Profiling. *Int. J. Mol. Sci.* 16: 23057-23075. doi: 10.3390/ijms160923057

Hu, R., Fan, C., Li, H., Zhang, Q., Fu, Y.F. (2009). Evaluation of putative reference genes for gene expression normalization in soybean by quantitative real- time RT-PCR. *BMC* *Mol. Biol. 10: 93*. doi: 10.1186/1471-2199-10-93.

Joshi, N.A. and Fass, J.N. (2011). Sickle: A sliding-window, adaptive, quality-based trimming tool for FastQ files (Version 1.33) [Software]. Available at https://github.com/najoshi/sickle.

Langmead, B., and Salzberg, S. (2012). Fast gapped-read alignment with Bowtie 2. *Nat. Methods* 9:357-359. doi: 10.1038/nmeth.1923

Liao, Y., Smyth, G.K., Shi, W. (2013). The Subread aligner: fast, accurate and scalable read mapping by seed-and-vote. *Nucleic Acids Res.* 41: e108. doi: 10.1093/nar/gkt214.

Love, M., Anders, S., Huber, W. (2014). Differential analysis of count data—the DESeq2 package. *Genome Biol.* 15:550. doi: 10.1186/s13059-014-0550-8

Luo, W., and Brouwer C. (2013). Pathview: an R/Bioconductor package for pathway-based data integration and visualization. *Bioinformatics* 29: 1830-1831. doi: 10.1093/bioinformatics/btt285

Oxborough, K. and Baker, N.R. (1997). An instrument capable of imaging chlorophyll a fluorescence from intact leaves at very low irradiance and at cellular and subcellular levels of organization. *Plant Cell Environ*. 20: 1473–1483. doi: 10.1046/j.1365-3040.1997.d01-42.x

Pimenta, M.R., Silva, P.A., Mendes, G.C., Alves, J.R., Caetano, H.D., Machado, J.P., Brustolini, O.J., Carpinetti, P.A., Melo, B.P., Silva, J.C., Rosado, G.L., Ferreira, M.F., Dal-Bianco, M., Picoli, E.A., Aragão, F.J., Ramos, H.J., Fontes, E.P.B. (2016). The Stress-Induced Soybean NAC Transcription Factor GmNAC081 Plays a Positive Role in Developmentally Programmed Leaf Senescence. *Plant Cell Physiol*. 57: 1098–1114. doi: 10.1093/pcp/pcw059

R Core Team (2020). R: A language and environment for statistical computing. R Foundation for Statistical Computing, Vienna, Austria.URL https://www.R-project.org/.

Weatherley, P.E. (1950). Studies in the water relations of the cotton plant. I. The field measurement of water deficits in leaves. *New Phytol.* 49: 81-97. doi: 10.1111/j.1469-8137.1950.tb05146.x

Weigel, D., and Glazebrook, J. (2002) Arabidopsis, a Laboratory Manual. New York: Cold Spring Harbour Press.

Wellburn, A.R. (1994). The spectral determination of chlorophylls a and b, as well as total carotenoids, using various solvents with spectrometers of different resolution. *J. Plant Physiol*. 144:307–313. doi: 10.1016/S0176-1617(11)81192-2
